# Patient-Reported Barriers to Adherence to Antiretroviral Therapy: A Systematic Review and Meta-Analysis

**DOI:** 10.1371/journal.pmed.1002183

**Published:** 2016-11-29

**Authors:** Zara Shubber, Edward J. Mills, Jean B. Nachega, Rachel Vreeman, Marcelo Freitas, Peter Bock, Sabin Nsanzimana, Martina Penazzato, Tsitsi Appolo, Meg Doherty, Nathan Ford

**Affiliations:** 1 Department of Infectious Disease Epidemiology, Imperial College London, London, United Kingdom; 2 Precision Global Health, Vancouver, Canada; 3 Department of Epidemiology, Infectious Diseases and Microbiology, University of Pittsburgh, Graduate School of Public Health Pittsburgh, Pennsylvania, United States of America; 4 Department of Medicine and Centre for Infectious Diseases, Stellenbosch University, Cape Town, South Africa; 5 Department of Epidemiology and International Health, Johns Hopkins Bloomberg School of Public Health, Baltimore, Maryland, United States of America; 6 Children's Health Services Research, Department of Pediatrics, Indiana University School of Medicine, Indianapolis, Indiana, United States of America; 7 Academic Model Providing Access to Healthcare (AMPATH), Eldoret, Kenya; 8 Department of HIV/AIDS, Ministry of Health, Brasilia, Brazil; 9 Desmond Tutu TB Centre, Department of Paediatrics and Child Health, Stellenbosch University, Stellenbosch, South Africa; 10 HIV, STI, and Other Blood Borne Infections Division, Rwanda Biomedical Centre, Ministry of Health, Rwanda; 11 University of Basel, Swiss Tropical and Public Health Institute, and Institute for Clinical Epidemiology and Biostatistics, Basel, Switzerland; 12 Department of HIV, World Health Organization, Geneva, Switzerland; 13 AIDS and TB Unit, Ministry of Health and Child Welfare, Harare, Zimbabwe; 14 Centre for Infectious Disease Epidemiology and Research, University of Cape Town, Cape Town, South Africa; University of California San Francisco School of Medicine, UNITED STATES

## Abstract

**Background:**

Maintaining high levels of adherence to antiretroviral therapy (ART) is a challenge across settings and populations. Understanding the relative importance of different barriers to adherence will help inform the targeting of different interventions and future research priorities.

**Methods and Findings:**

We searched MEDLINE via PubMed, Embase, Web of Science, and PsychINFO from 01 January 1997 to 31 March 2016 for studies reporting barriers to adherence to ART. We calculated pooled proportions of reported barriers to adherence per age group (adults, adolescents, and children). We included data from 125 studies that provided information about adherence barriers for 17,061 adults, 1,099 children, and 856 adolescents. We assessed differences according to geographical location and level of economic development. The most frequently reported individual barriers included forgetting (adults 41.4%, 95% CI 37.3%–45.4%; adolescents 63.1%, 95% CI 46.3%–80.0%; children/caregivers 29.2%, 95% CI 20.1%–38.4%), being away from home (adults 30.4%, 95% CI 25.5%–35.2%; adolescents 40.7%, 95% CI 25.7%–55.6%; children/caregivers 18.5%, 95% CI 10.3%–26.8%), and a change to daily routine (adults 28.0%, 95% CI 20.9%–35.0%; adolescents 32.4%, 95% CI 0%–75.0%; children/caregivers 26.3%, 95% CI 15.3%–37.4%). Depression was reported as a barrier to adherence by more than 15% of patients across all age categories (adults 15.5%, 95% CI 12.8%–18.3%; adolescents 25.7%, 95% CI 17.7%–33.6%; children 15.1%, 95% CI 3.9%–26.3%), while alcohol/substance misuse was commonly reported by adults (12.9%, 95% CI 9.7%–16.1%) and adolescents (28.8%, 95% CI 11.8%–45.8%). Secrecy/stigma was a commonly cited barrier to adherence, reported by more than 10% of adults and children across all regions (adults 13.6%, 95% CI 11.9%–15.3%; children/caregivers 22.3%, 95% CI 10.2%–34.5%). Among adults, feeling sick (15.9%, 95% CI 13.0%–18.8%) was a more commonly cited barrier to adherence than feeling well (9.3%, 95% CI 7.2%–11.4%). Health service–related barriers, including distance to clinic (adults 17.5%, 95% CI 13.0%–21.9%) and stock outs (adults 16.1%, 95% CI 11.7%–20.4%), were also frequently reported. Limitations of this review relate to the fact that included studies differed in approaches to assessing adherence barriers and included variable durations of follow up. Studies that report self-reported adherence will likely underestimate the frequency of non-adherence. For children, barriers were mainly reported by caregivers, which may not correspond to the most important barriers faced by children.

**Conclusions:**

Patients on ART face multiple barriers to adherence, and no single intervention will be sufficient to ensure that high levels of adherence to treatment and virological suppression are sustained. For maximum efficacy, health providers should consider a more triaged approach that first identifies patients at risk of poor adherence and then seeks to establish the support that is needed to overcome the most important barriers to adherence.

## Abstract 2

In this systematic review and meta-analysis, Nathan Ford and colleagues assess the most frequently reported barriers by patients experiencing challenges adhering to antiretroviral therapy.

## Author Summary

**Why Was This Study Done?:**

- Despite more than two decades of research on adherence to antiretroviral therapy (ART) and more than 17 million HIV-positive individuals on treatment, adherence to ART remains a major challenge.
- This review aimed to assess the most frequently reported barriers to adherence by patients experiencing adherence challenges.

**What Did the Researchers Do and Find?:**

- Published data from 125 studies on patient-reported barriers to adherence were systematically reviewed and analyzed by age group.
- The most frequently reported individual barriers across all age groups included forgetting, being away from home, and a change to daily routine. Depression was reported as a barrier to adherence by more than 15% of patients across all age categories, while alcohol/substance misuse was commonly reported as a barrier by adults and adolescents.
- With respect to contextual barriers, secrecy/stigma was a commonly cited barrier to adherence, reported by more than 10% of patients across all regions.
- Health service–related barriers were frequently reported, including distance to clinic and stock outs.

**What Do These Findings Mean?:**

- Evidence-based interventions exist that may address many of the most common patient-reported barriers to adherence.
- However, no single intervention will be sufficient to ensure that high levels of adherence to treatment and virological suppression are sustained.
- Health providers should consider a more triaged approach that first identifies patients at risk of poor adherence and then seeks to establish the support that is needed to overcome the most important barriers to adherence.
- Several key health service improvements are also required to ensure that patients are able to consistently access ART.

## Introduction

Global targets for scaling up antiretroviral therapy (ART) include ensuring that 90% of patients on ART achieve viral suppression. This gives a renewed emphasis to ensuring optimal levels of adherence. Negative outcomes of longer-term suboptimal adherence include increased risk of disease progression [1], drug resistance [2], high viral load and consequent risk of transmission [3,4], and death [5,6].

Maintaining high levels of adherence is a challenge across settings. Suboptimal adherence to antiretroviral medication has been reported for specific patient groups such as adolescents [7], pregnant women [8], and others in high-, middle-, and low-income countries. A broad range of context-specific barriers to adherence have been reported, including forgetfulness, stigma, adverse drug reactions, and competing responsibilities [9,10]. These challenges have been categorized as individual, interpersonal, community, and structural factors [11].

Several interventions have been found to improve adherence in randomized trials, including adherence counselling, text messaging, and reminder devices [12], and these are recommended by the WHO [13]. However, there remains a need to understand the relative importance of different barriers to adherence in order to inform the targeting of different interventions and inform future research.

We conducted this systematic review to assess patient reported barriers to adherence among HIV-infected adults, adolescents and children in high-, middle-, and low-income countries.

## Methods

### Search Strategy and Selection Criteria

This study follows the Preferred Reporting Items for Systematic Reviews and Meta-Analysis (PRISMA) statement [14]. The study protocol and PRISMA statement are available in the Supporting Information (S1 Text and S2 Text) [14].

To be included, studies had to provide information about barriers to adherence reported by at least 50 adult patients or 20 children or their caregivers who were non-adherent to ART according to study definitions. These cut offs were chosen in practical consideration of the high number of studies identified through preliminary searches and the limited and sometimes unreliable information to be gained from small studies [15]. Using a search strategy that combined terms for ART, adherence, and commonly reported reasons for non-adherence (See S1 Text), two investigators (ZS, NF) screened MEDLINE via PubMed, Embase, Web of Science, and PsychINFO from 01 January 1997 to 31 March 2016. We also screened abstracts from all International AIDS Society conferences, all conferences on HIV Treatment and Prevention Adherence, and all conferences of the European Society for Patient Adherence, Compliance, and Persistence from 2012 to 2015, and electronically available abstracts of the Conference on Retroviruses and Opportunistic Infections (2014–2016) to identify recent studies that may not yet have been published in full. We supplemented database searches by screening bibliographies of review articles and all included full-text articles. Studies that included patients mainly receiving dual- or mono-therapy (>20% of cohort), antiretroviral interventions other than treatment (PEP, PrEP, or PMTCT), or reported adherence to medication other than ART were excluded from review. We only extracted data on non-adherent patients, following the definition of non-adherence provided by the studies; if studies included both adherent and non-adherent patients and data could not be disaggregated, the study was excluded. Studies assessing adherence interventions were also excluded unless baseline (i.e., pre-intervention) information was relevant to our analysis. Qualitative studies were excluded unless relevant quantitative data were also provided. No language restriction was applied.

### Data Extraction and Analysis

We extracted data independently in duplicate (ZS, NF) using a standardized, piloted data extraction form and following the predefined protocol. Barriers to adherence were initially categorized according to AIDS Clinical Trials Group adherence instrument items, with additional categories developed to capture challenges common in resource-limited settings [16]. Using categories adapted from a qualitative review of adherence barriers [11], responses were grouped into individual, contextual, and health service–related barriers; where there was uncertainty, investigators discussed to achieve consensus. Information about study design and setting, age of patients, and adherence measure was also extracted. The following variables were extracted to assess study quality: use of a previously piloted and/or validated questionnaire to assess barriers to adherence; random sampling; and use of objective adherence measures. These indicators were identified following a review of the first ten eligible studies.

We calculated proportions and corresponding 95% CIs for all reported barriers to adherence and pooled data following transformation [17,18], using random effects models stratified by age (adults, adolescents, and children, as defined by the studies) [19]. Because statistical tests for heterogeneity are not reliable for pooled proportions [20], we assessed heterogeneity through visual inspection of forest plots. We ran prespecified subgroup analyses to assess the potential influence of study quality indicators and explored changes over time (assessed using date of study completion) using meta-regression. All analyses were conducted in Stata version 13.0.

## Results

**Fig 1:**
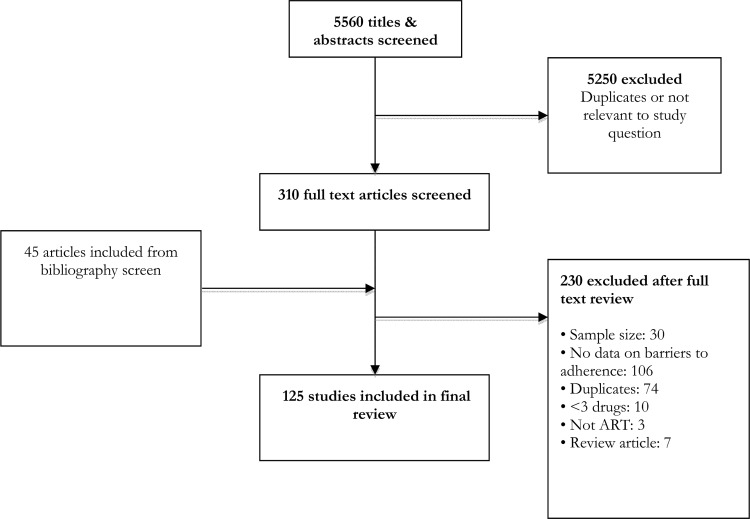
Study selection process.

From an initial screen of 5,560 abstracts, 125 studies met our inclusion criteria (Fig 1). These studies provided information about barriers to adherence for 19,016 patients—17,061 adults, 1,099 children, and 856 adolescents—with documented non-adherence to ART. Studies were carried out across 38 countries, with the majority carried out in the Africa region (58 studies, 16 countries), the European region (14 studies, 10 countries), and the Western Pacific region (8 studies, 6 countries). Study quality was rated to be moderate overall. The majority of studies (78/125, 62%) used a validated questionnaire to assess barriers to adherence and piloted the questionnaire (89/125, 71%); however, less than half of studies (38/125, 30%) used random sampling, and objective adherence measures (pill count, pharmacy refill, and viral load) were only used in the minority (18/125, 14%) of studies; these limitations are important potential sources of selection and information bias. The most common definitions of adherence were no missed doses (55 studies) and >95% adherence (37 studies). Average duration on ART ranged from 4 wk to 239 wk (median 78 wk). Characteristics of included studies are summarized in S1 Table.

**Fig 2:**
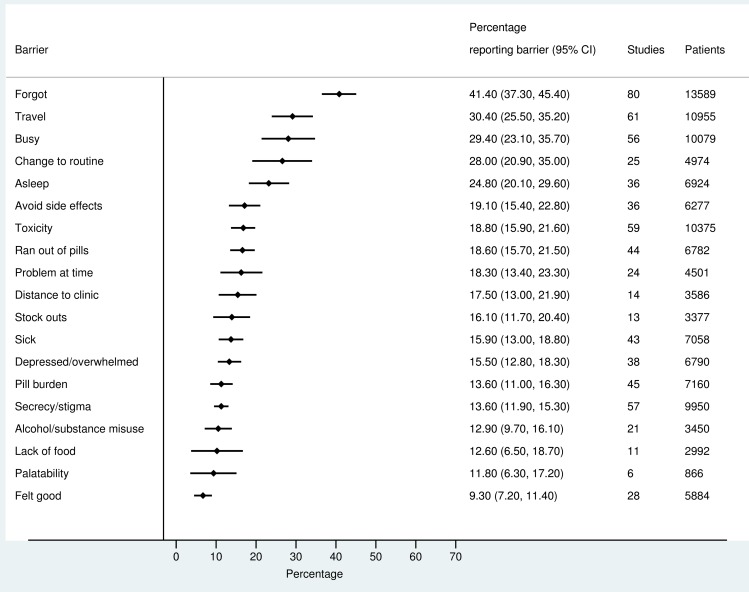
Barriers to adherence among adults on ART.

**Fig 3:**
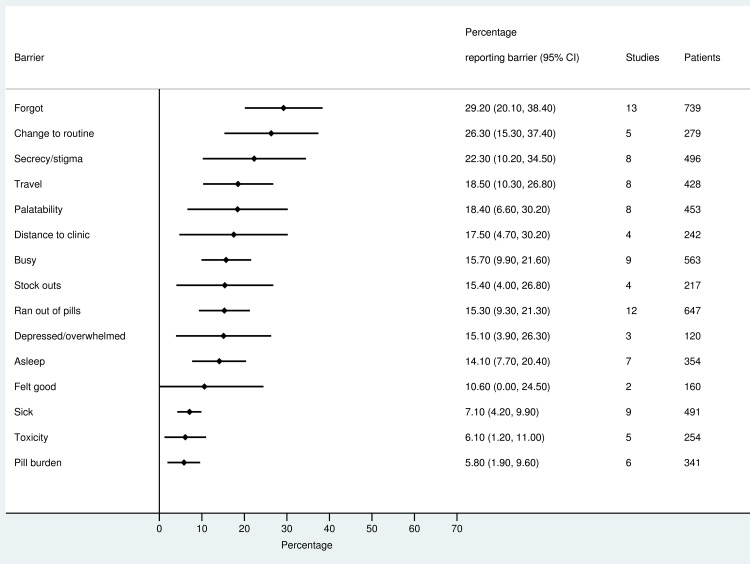
Barriers to adherence among children on ART.

**Fig 4:**
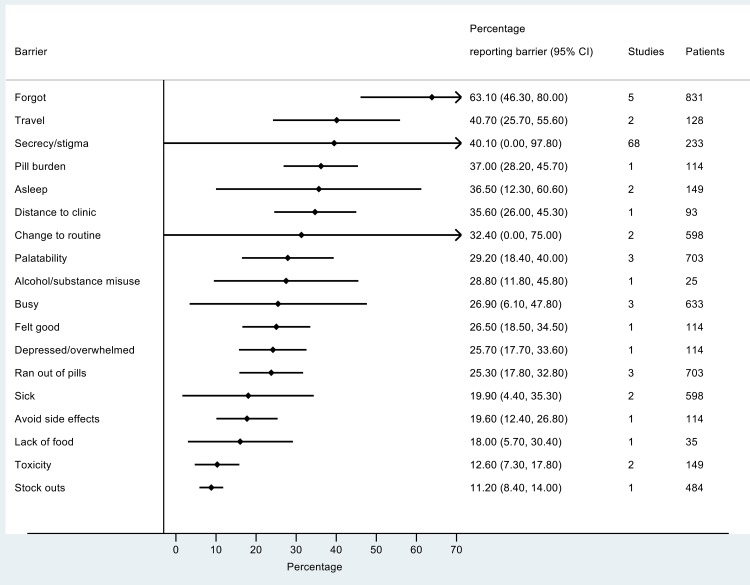
Barriers to adherence among adolescents on ART.

The most frequently reported barriers to adherence for adults, adolescents, and children are summarized in Figs 2–4. The most frequently reported individual barriers across all age groups included forgetting, being away from home, and a change to daily routine. Depression was reported as a barrier to adherence by more than 15% of patients across all age categories, while alcohol/substance misuse was commonly reported as a barrier by adults and adolescents. Among adults, feeling sick was a more commonly cited barrier to adherence than feeling well (relative risk 1.68, 95% CI 1.23–2.30). The proportion of adolescents reporting barriers to adherence was higher per barrier compared to adults, but data are limited and confidence intervals are wide for most estimates.

With respect to contextual barriers, secrecy/stigma was a commonly cited barrier to adherence, reported by more than 10% of patients across all regions (S2 Table). Notably, secrecy/stigma was more commonly reported as a barrier to adherence among children/caregivers compared to adults.

Health service–related barriers were frequently reported, including distance to clinic and stock outs. Distance to clinic was reported as a barrier for all age groups across 11 low- and middle-income countries in the Africa, South East Asia, and Western Pacific regions. Stock outs were reported across 13 countries for adults, all in low- and middle-income countries. For children, two studies reported stock outs in the United States. Barriers related to drug toxicity were frequently reported among adults and adolescents, while for children and adolescents, palatability was an important concern. In meta-regression, there was evidence that the frequency of reporting toxicity, pill burden, and being sick as barriers to adherence have reduced over time (Table 1).

**Table 1:** Random effects meta-regression.

Covariate	Outcome	β coefficient	95% CI	*p*-value
Study date	Toxicity	-0.009	-0.017–0	0.045
	Side effects	-0.007	-0.018–0.03	0.162
	Pill burden	-0.009	-0.017–0	0.045
	Palatability	-0.012	-0.003–0.050	0.16
	Distance to clinic	-0.003	-0.040–0.320	0.834
	Secrecy/stigma	0.001	-0.009–0.010	0.908
	Cost	0	-0.052–0.010	0.115
	Felt sick	-0.011	-0.186–-0.030	0.006

## Discussion

Our review highlights the diversity of patient-reported barriers to adherence across age groups. The most frequently reported individual barriers across all age groups included forgetting, being away from home, depression, and a change to daily routine; alcohol and/or substance misuse was commonly reported by adults and adolescents. Health service–related barriers, including distance to clinic and stock outs, were also frequently reported.

Most barriers to adherence are amenable to interventions that have been evaluated in randomized trials (Table 2) [12,21–30]. Notably, forgetting was the most frequently cited barrier to adherence across all age groups. Challenges relating to timing of medication, including being asleep, could be overcome through text messaging, reminder devices, and individual counseling that seeks to routinize medication taking in a way that fits in with other daily activities [31].

**Table 2:** Common barriers to adherence and corresponding interventions supported by evidence from randomized trials.

Barrier	Intervention
Forgetfulness	SMS messaging; reminder devices [12]
Travel	Pillbox organizers [21]
Toxicity	ART regimens with reduced toxicity [22]
Distance to clinic	Decentralization of services [23]; reduced frequency of clinic visits [24]
Depression	Mental health screening and support [12, 26–28]
Alcohol/substance misuse	Counselling [12, 25]
Palatability	Improved pediatric formulations [29]
Pill burden	Fixed-dose combinations [30]

We found that health service barriers played an important role in frustrating efforts to maintain high levels of adherence to treatment. Long distance to clinics is a risk factor for loss to care [32], and decentralization of HIV services is associated with better retention [23]. Recent stock outs of antiretroviral medication have been recorded in several countries in Africa [33], and there is a pressing need for increased vigilance as countries move to adopt the policy of treating all HIV-positive individuals and consider transitioning from established first-line medications to newer regimens.

Previously, concern has been expressed that people who receive ART early in their disease progression may be less adherent to treatment [34,35]. The finding that feeling sick was a more commonly reported barrier to adherence than feeling well suggests that this may not be the case and supports the recent recommendation by WHO to treat all HIV-positive individuals regardless of immune status [36]. As HIV programs start to provide early ART to people earlier in their HIV infection, it will be important to prospectively collect data to further evaluate this concern.

Toxicity and pill burden have both been found to be associated with poor adherence in other reviews [37,38]. This review found that the frequency of reporting these factors as barriers to adherence has reduced over time, which is consistent with efforts by WHO and other agencies to promote fixed-dose combinations and rationalize treatment guidelines towards the use of antiretroviral drugs associated with a better safety profile [39].

The main adherence barriers identified by our review are consistent with a recent review by Langenbeek et al that found substance use, concerns about ART, satisfaction with care providers, stigma, social support, and self-efficacy to be strongly associated with adherence [40]. In contrast to the Langenbeek review, which assessed the influence between baseline patient characteristics and adherence, our review assesses adherence barriers that are reported by patients and, as such, was able to identify a number of additional frequently reported barriers that could not be gleaned from clinic records; because of this difference in approach, the number of studies in our review that were included in the previous review is small (19%). This approach builds on a previous review of patient-reported barriers to adherence that was published in 2006 [10]. There have been considerable changes in ART delivery over the past decade: the number of patients on ART globally has increased; drug regimens have improved with respect to tolerability and simplicity; and service provision has been decentralized. In updating this review, we have been able to include a larger sample size that allowed for a ranking in the frequency of reporting of barriers, disaggregated by age, and an understanding of how these barriers differ by geographical region and over time. This can allow for a better understanding about where resources need to be focused in order to improve adherence among different patient populations.

Several recent studies have indicated that adolescents face challenges across the continuum of HIV care, and outcomes of ART are worse for adolescents compared to adults [41]. HIV programs should pay particular attention to the adherence challenges faced by this vulnerable population and target adherence interventions accordingly; such an approach would be facilitated by the development of better ways to measure adherence. To date, few adolescents have been enrolled into trials of interventions to improve adherence, and this is an important area of future research [42]. A pilot feasibility study found that personalized, interactive, daily text message reminders were feasible and acceptable, and significantly improved self-reported adherence. However, larger controlled studies are needed to determine the impact of this intervention on ART adherence and other related health outcomes for youth living with HIV/AIDS globally [43,44].

Pregnant and postpartum women are another group who face challenges in maintaining high levels of adherence to medication [8]. Despite the critical need for ART during pregnancy and the postpartum period, evidence-based interventions to promote ART adherence during this period are lacking. A recent exploratory study of 109 HIV-positive pregnant South African women found that mobile phone access (>90%) and interest in text messaging for adherence support (88.1%) was high, and the majority (95%) of women were willing to disclose their status to a treatment buddy/supporter [45].

More generally, the fact that most adherence intervention studies are only able to show a modest effect in randomized trials is likely in part a consequence of the multiple challenges patients face in adhering to treatment as indicated by the findings of our review (i.e., within studies the percentages of reported barriers added up to more than 100%). Future research is encouraged to evaluate the effectiveness (effect size and interaction effects) of more than one intervention on virological suppression, using a factorial or adaptive clinical trial design to precisely determine the specific interventions and components of interventions that work best. [46].

Our review has several strengths and limitations. Strengths include our broad search strategy and inclusion criteria that allowed for the identification of a substantial number of studies and synthesis of a large dataset. Limitations are mainly related to study quality and include the variable definitions of adherence used by the different studies, different approaches to assessing adherence barriers and time on ART and it is possible that these and other unreported factors may have influenced outcomes. Information about drug toxicity is limited by the possibility that not all experienced adverse events are related to ART, even if they were perceived as a reason to stop taking the medication. Caution is also needed in the interpretation of results as some reasons for poor adherence (e.g., forgetting) may be put forward because they are perceived to be more socially acceptable than others (e.g., chaotic lifestyle or substance misuse). Although several analyses were undertaken to identify potential explanations for variance in findings, we could not thoroughly explore all possible differences in covariates (e.g., geographic region, income) due to the need to avoid spurious associations that may arise from large numbers of outcomes and covariates. We searched multiple databases and conferences, which allowed us to include data from over 100 studies for analysis; however, we did not include regional databases, and this may have limited identification of potentially eligible studies. An additional limitation to note with respect to children is that barriers were mainly reported by caregivers, and these may not represent the most important barriers faced by children themselves [47]. Finally, any study that looks at self-reported adherence will likely underestimate the frequency of non-adherence, and studies that assessed objective measures of adherence are more likely to be accurate in terms of reflecting true adherence rates.

In conclusion, this review highlights that patients on ART face multiple barriers to adherence and no single intervention will be sufficient to ensure that high levels of adherence to treatment and virological suppression are sustained. Rather than introducing single interventions into HIV programs, health providers should consider a more triaged approach that first identifies patients at risk of poor adherence and then seeks to establish the support that is needed to overcome the most important barriers to adherence. For maximum efficacy, adherence support strategies should be targeted to those individuals who require support. Finally, although the majority of the most commonly reported barriers are amenable to intervention at the individual level, several key health service improvements are also required to ensure that patients are able to access ART.

## Supporting Information

S1 Table Characteristics of included studies. (DOCX)

S2 Table Individual meta-analysis for each barrier, by age and geographical region. (DOCX)

S1 Text Systematic review protocol. (DOCX)

S2 Text PRISMA checklist. (DOC)

## Abbreviations

ART: antiretroviral therapy
PRISMA: Preferred Reporting Items for Systematic Reviews and Meta-Analysis
