# Supplementary material for: Patient-Reported Barriers to Adherence to Antiretroviral Therapy: A Systematic Review and Meta-Analysis
Source: PLoS Med. 2016 Nov 29;13(11):e1002183. doi: 10.1371/journal.pmed.1002183 (PMC5127502; doi:10.1371/journal.pmed.1002183)
Supplement: S1 Table — (DOCX) [file pmed.1002183.s001.docx]

**Table S1: Characteristics of included studies**

| **Author** | **WHO region** | **Income group** | **Year of Publication** | **Study end** | **Country** | **Adherence definition** | **Weeks on ART** | **Recall period** | **Age** | **Number non adherent** | **Adherence measure** | **Random sampling*** | **Questionnaire*** | |
| --- | --- | --- | --- | --- | --- | --- | --- | --- | --- | --- | --- | --- | --- | --- |
|  |  |  |  |  |  |  |  |  |  |  |  |  | **Piloted** | **Validated** |
| Achappa^1^ | SEARO | LMIC | 2013 | 2013 | India | >95% | 52 | 4 days | Adults | 46 | Self report | No | Yes | Yes |
| Aderemi-Williams^2^ | AFRO | LMIC | 2012 | 2012 | Nigeria | NR | NR | 7 day | Children | 40 | Self report | Yes | No | No |
| Alakija Kazeem^3^ | AFRO | LMIC | 2010 | 2009 | Nigeria | >95% | 156 (median); 26 (minimum) | 1 month | Adults | 74 | Self report | No | No | No |
| Amberbir^4^ | AFRO | LMIC | 2008 | 2007 | Ethiopia | >95% | 35 | 7 days | Adults | 87 | Self report | No | Yes | Yes |
| Amico^5^ | AMRO N | HIC | 2007 | 2005 | USA | No missed doses | NR | 3 days | Adults | 72 | Self report | No | Yes | Yes |
| Ammassari^6^ | EURO | HIC | 2001 | 2000 | Italy | No missed doses | 52 | 3 days | Adults | 79 | Self report | No | Yes | Yes |
| Angevine^7^ | AFRO | LMIC | 2007 |  | Uganda | No missed doses | 26 | Ever | Adolescents | 35 | Self report | No | Yes | Yes |
| Arage^8^ | AFRO | LMIC | 2014 | 2013 | Ethiopia | >95% | 4 | 1 month | Children | 94 | Self report | No | Yes | Yes |
| Arrondo-Velasco^9^ | EURO | HIC | 2009 | 2005 | Spain | >90% | 13 | 3 months | Adults | 149 | Self report | Yes | Yes | Yes |
| Babatunde^10^ | AFRO | LMIC | 2013 | 2013 | Nigeria | NR | 60 | 7 day | Adults | 40 | Self report | Yes | No | No |
| Baltazary^11^ | AFRO | LMIC | 2011 | 2011 | Tanzania | NR | NR |  | Adults | 197 |  | No | No | No |
| Bant^12^ | SEARO | LMIC | 2013 | 2012 | India | >95% | 24 |  | Adults | 90 |  | No | No | No |
| Barfod^13^ | EURO | LMIC | 2006 | 2003 | Denmark | No missed doses | 270 | 4 days | Adults | 127 | Self report | Yes | Yes | Yes |
| Belayneh^14^ | AFRO | LMIC | 2015 | 2014 | Ethiopia | NR | NR | 4 days | Adults | 169 | Self report | No | No | No |
| Bello^15^ | AFRO | LMIC | 2011 | 2010 | Nigeria | >95% | 26 | 1 month | Adults | 62 | Self report, pill count | No | Yes | Yes |
| Bhat^16^ | AFRO | LMIC | 2010 | 2009 | South Africa | No missed doses | NR | Ever | Adults | 63 | Self report | No | Yes | No |
| Biadgilign^17^ | AFRO | LMIC | 2008 | 2008 | Ethiopia | >95% | 104 | 7 days | Children | 51 | Self report | Yes | Yes | Yes |
| Biressaw^18^ | AFRO | LMIC | 2013 | 2012 | Ethiopia | >95% | 208 | Ever | Children | 70 | Self report | No | Yes | Yes |
| Blanco^19^ | EURO | HIC | 2005 | 2002 | Spain | No missed doses | NR | 3 months | Adults | 154 | Self report | No | Yes | Yes |
| Buchanan^20^ | AMRO N | HIC | 2012 | 2007 | USA | No missed doses | NR | 1 month | Children | 73 | Self report | Yes | Yes | No |
| Byakika-Tusiime^21^ | AFRO | LMIC | 2005 | 2002 | Uganda | No missed doses | 30-52 (median); 4 (minimum) | 3 months | Adults | 134 | Self report | No | No | No |
| Chesney^22^ | AMRO N | HIC | 2000 | 1997 | USA | No missed doses | NR | Ever | Adults | 51 | Self report | No | Yes | Yes |
| Cook^23^ | AMRO N | HIC | 2001 | 1998 | USA | No missed doses | NR | 7 days | Adults | 217 | Self report | No | Yes | Yes |
| Crozatti^24^ | AMRO S | LMIC | 2013 | 2013 | Brazil | >90% | NR | 4 days | Adolescents | 105 | Self report | No | Yes | Yes |
| Cummings^25^ | WPRO | HIC | 2003 | 2003 | Australia | No missed doses | NR | Ever | Adults | 56 | Self report | No | Yes | No |
| Dachew^26^ | AFRO | LMIC | 2014 | 2012 | Ethiopia | >95% | 8 | Unclear | Children | 84 | Self report | Yes | Yes | Yes |
| Dashe^27^ | AFRO | LMIC | 2007 |  | Nigeria | >95% | NR | Ever | Adults | 1067 | Self report | Yes | Yes | Yes |
| Davies^28^ | AFRO | LMIC | 2008 | 2004 | South Africa | No missed doses | 13 | 3 months | Children | 33 | Caregiver | Yes | Yes | Yes |
| DeMayo^29^ | AMRO N | HIC | 2012 |  | USA | No missed doses | NR | 30 days | Adults | 168 | Self report | No | No | Yes |
| Do^30^ | WPRO | LMIC | 2011 | 2009 | Vietnam | No missed doses | 80 | Ever | Adults | 615 | Self report | No | Yes | Yes |
| Dyrehave^31^ | AFRO | LMIC | 2015 | 2013 | Guinee Bissau | No missed doses | 12 | 4 days | Adults | 494 | Self report | No | Yes | Yes |
| Egieyeh^32^ | AFRO | LMIC | 2008 | 2007 | Nigeria | NR | NR | 1 month | Adults | 97 | Self report, pill count | No | No | No |
| Ehlers^33^ | AFRO | LMIC | 2015 | 2012 | Botswana | >95% | 26 | 4 days | Adults | 57 | Pharmacy refill | Yes | No | Yes |
| Eholie^34^ | AFRO | LMIC | 2007 | 2002 | Cote d'Ivoire | >90% | 83 | 7 days | Adults | 235 | Self report | Yes | Yes | No |
| Ejekam^35^ | AFRO | LMIC | 2014 | 2014 | Nigeria | No missed doses | 26 | 3 days | Adults | 198 | Self report | Yes | No | No |
| Elise^36^ | AFRO | LMIC | 2005 | 2004 | Cote d'Ivoire | No missed doses | 140 | 1 month | Children | 37 | Self report | Yes | Yes | No |
| Essomba^37^ | AFRO | LMIC | 2015 | 2014 | Cameroun | >95% | NR | 30 days | Adults | 257 | Self report | Yes | Yes | Yes |
| Eticha^38^ | AFRO | LMIC | 2014 | 2013 | Ethiopia | >95% |  | 7 days | Children | 32 | Self report | Yes | Yes | Yes |
| Fredericksen^39^ | AMRO N | HIC | 2013 | 2013 | USA | NR | NR | 30 days | Adults | 42 | Self report | No | No | No |
| Gare^40^ | WPRO | LMIC | 2015 | 2011 | Papua New Guinea | >95% | 80 | Ever | Adults | 47 | Self report, pill count | No | No | No |
| Genberg^41^ | AMRO N | HIC | 2015 | 2005 | USA | No missed doses | 81 | 30 days | Adults | 503 | Self report, MEMS | No | Yes | Yes |
| Gibb^42^ | EURO | HIC | 2003 | 1999 | Europe and Brazil | No missed doses | 48 | . | Children | 65 | Self report | No | Yes | No |
| Gifford^43^ | AMRO N | HIC | 2000 | 2000 | USA | No missed doses | NR | 7 days | Adults | 67 | Self report | No | Yes | Yes |
| Gir^44^ | AMRO S | LMIC | 2005 | 2003 | Brazil | NR | 286 | Ever | Adults | 156 | Self report | Yes | No | Yes |
| Gokarn^45^ | SEARO | LMIC | 2012 | 2009 | India | >95% | 26 | 1 month | Adults | 86 | Self report | Yes | Yes | No |
| Golin^46^ | AMRO N | HIC | 2002 | 1999 | USA | No missed doses | 104 | Ever | Adults | 71 | Self report | No | Yes | Yes |
| Guimarães^47^ | AMRO S | LMIC | 2008 |  | Brazil | NR | 30 | Ever | Adults | 78 | Self report | No | No | Yes |
| Hansana^48^ | WPRO | LMIC | 2013 | 2011 | Laos | >95% | 166 | Ever | Adults | 135 | Self report | Yes | Yes | Yes |
| Haberer^49^ | AFRO | LMIC | 2012 | 2012 | Uganda | NR | NR | 2 day | Adults | 64 | Wisepill | No | No | No |
| Harris^50^ | EURO | LMIC | 2011 | 2005 | Dominican Republic | >95% | NR | 1 month | Adults | 71 | Self report | No | No | Yes |
| Hardon^51^ | AFRO | LMIC | 2006 | 2005 | Botswana | >95% | 39 | 1 month | Adults | 201 | Self report | No | No | No |
| Harvey^52^ | AMRO S | LMIC | 2008 | 2006 | Jamaica | >95% | NR | 7 days | Adults | 116 | Self report | Yes | Yes | Yes |
| Heckman^53^ | AMRO N | HIC | 2004 | 2002 | USA | No missed doses | NR | ever missed | Adults | 127 | Self report | No | Yes | Yes |
| Igwebe^54^ | AFRO | LMIC | 2010 | 2010 | Nigeria | >95% | 182 | 1 month | Adults | 80 | Self report | No | No | Yes |
| Ilyaso^55^ | AFRO | LMIC | 2005 | 2005 | Nigeria | No missed doses | NR | . | Adults | 202 | Self report | No | No | Yes |
| Iroha^56^ | AFRO | LMIC | 2010 | 2008 | Nigeria | No missed doses | 78 | 3 days | Children | 29 | Self report | No | No | Yes |
| Jean-Baptiste^57^ | AFRO | LMIC | 2008 | 2005 | Rwanda | No missed doses | 26 | 1 month | Adults | 163 | Self report | Yes | Yes | Yes |
| Joshi^58^ | SEARO | LMIC | 2008 | 2008 | India | No missed appointment | NR | . | Adults | 93 | Self report | No | No | No |
| Kalichman^59^ | AMRO N | HIC | 1999 | 1999 | USA | No missed doses | NR | 30 days | Adults | 70 | Self report | No | Yes | No |
| Kerr^60^ | AMRO N | HIC | 2004 | 2002 | Canada | >95% | NR | Ever | Adults | 108 | Self report, pharmacy record | No | Yes | Yes |
| Kleeberger^61^ | AMRO N | HIC | 2001 | 1999 | USA | >100% | 26 | 4 days | Adults | 120 | Self report | No | Yes | Yes |
| Koole^62^ | AFRO | LMIC | 2016 | 2011 | Tanzania, Uganda and Zambia | No missed doses | 208 | Ever | Adults | 1278 | Self report | Yes | Yes | Yes |
| Lal^63^ | SEARO | LMIC | 2010 | 2005 | India | >95% | NR | Ever | Adults | 60 | Self report | No | Yes | Yes |
| Letta^64^ | AFRO | LMIC | 2015 | 2015 | Ethiopia | No missed doses | 24 | 7 days | Adults | 93 | Self report | Yes | Yes | Yes |
| MacDonell^65^ | AMRO N | HIC | 2013 | 2013 | USA | No missed doses | NR | 7 days | Adolescents | 484 | Self report | No | Yes | Yes |
| Marhefka^66^ | AMRO N | HIC | 2004 | 2004 | USA | >90% | 361 | 3 months | Children | 51 | pharmacy refill | No | Yes | Yes |
| Marhefka^67^ | AMRO N | HIC | 2008 | 2003 | USA | >90% | NR | Ever | Children | 92 | Self report | Yes | Yes | Yes |
| Markos^68^ | AFRO | LMIC | 2008 | 2006 | Ethiopia | No missed doses | 44 | 7 days | Adults | 75 | Self report, pill count | Yes | Yes | Yes |
| Mavhu^69^ | AFRO | LMIC | 2013 | 2009 | Zimbabwe | No missed doses | NR | Ever | Adolescents | 93 | Self report | Yes | Yes | Yes |
| Mbopi-Kéou^70^ | AFRO | LMIC | 2012 | 2010 | Cameroun | >95% | 130 | Ever | Adults | 173 | Self report, pharmacy record | Yes | No | No |
| Mehta^71^ | SEARO | LMIC | 2015 | 2013 | India | >95% | 159 | 3 months | Children | 27 | Self report, viral load | No | Yes | No |
| Moatti^72^ | EURO | HIC | 2000 | 1998 | France | >80% | 23 | 7 days | Adults | 57 | Self report | No | No | No |
| Mohammed^73^ | AMRO N | HIC | 2004 | 2001 | USA | No missed doses | NR | 1 month | Adults | 214 | Self report | No | Yes | Yes |
| Monreal^74^ | AMRO S | LMIC | 2002 | 2000 | Brazil | >80% | 104 | 7 days | Adults | 93 | Self report | No | Yes | No |
| Moralejo^75^ | EURO | HIC | 2006 | 2006 | Spain | No missed doses | 77 | Ever | Adults | 117 | Self report | No | Yes | Yes |
| Morrison^76^ | EURO | HIC | 2014 | 2009 | Albania | >95% | NR | 4 days | Adults | 68 | Self report, pharmacy record | Yes | Yes | Yes |
| Murphy^77^ | AMRO N | HIC | 2003 | 2003 | USA | No missed doses | NR | 1 month | Adolescents | 114 | Self report | No | Yes | Yes |
| Naik^78^ | SEARO | LMIC | 2009 | 2009 | India | No missed doses | . | Ever | Adults | 65 | Self report | Yes | Yes | Yes |
| Nieuwkerk^79^ | EURO | HIC | 2011 | 1999 | Netherlands | No missed doses | 81 | 7 days | Adults | 105 | Self report | No | Yes | No |
| Nwauche^80^ | AFRO | LMIC | 2006 | 2005 | Nigeria | >95% | 26 | . | Adults | 95 | Self report | No | No | No |
| Nwokike^81^ | AFRO | LMIC | 2004 | 2002 | Botswana | >95% | 13 | 7 days | Adults | 75 | Self report, pill count | Yes | Yes | No |
| Nyaku^82^ | AMRO N | HIC | 2015 | 2011 | USA | NR | NR |  | Adults | 415 | Self-report | Yes | No | No |
| Nyogea^83^ | AFRO | LMIC | 2014 | 2012 | Tanzania |  | 120 | 3 day | Adults | 364 | Pill-count | Yes | No | No |
| Obirikorang^84^ | AFRO | LMIC | 2013 | 2013 | Ghana | No missed doses | 130 | Ever | Adults | 76 | Self report | No | Yes | No |
| Odili^85^ | AFRO | LMIC | 2016 | 2013 | Nigeria | No missed doses | 156 | 1 month | Adults |  | Self report | No | Yes | Yes |
| Okonsky^86^ | AMRO N | HIC | 2015 | 2011 | USA | >100% | NS | 1 month | Adults | 91 | Self report | No | Yes | Yes |
| Okoronkwo^87^ | AFRO | LMIC | 2013 |  | Nigeria | >95% | 52 | Ever | Adults | 188 | Self report, pill count | No | Yes | Yes |
| Oku^88^ | AFRO | LMIC | 2013 | 2011 | Nigeria | >95% | 104 | 7 days | Adults | 165 | Self report | No | Yes | Yes |
| Oku^89^ | AFRO | LMIC | 2014 | 2012 | Nigeria | >95% | 113 | 7 days | Adults | 195 | Self report | No | Yes | Yes |
| Olowookere^90^ | AFRO | LMIC | 2008 | 2007 | Nigeria | >95% | 82 | Ever | Adults | 216 | Self report | No | No | Yes |
| Pennap^91^ | AFRO | LMIC | 2013 | 2013 | Nigeria | No missed doses | 26 (minimum) | 7 days | Adults | 201 | Self report | No | No | No |
| Rao^92^ | AMRO N | HIC | 2007 | 2007 | USA | NR | NR | Ever | Adolescents | 25 | Self report | No | No | No |
| Remien^93^ | AMRO S | LMIC | 2007 | 2007 | Brazil | >80% | NR | Ever | Adults | 200 | Self report | No | Yes | Yes |
| Riera^94^ | EURO | HIC | 2002 | 1998 | Spain | >90% | 41 | 3 months | Adults | 59 | Self report, pill count, drug levels | No | Yes | Yes |
| Saberi^95^ | AMRO N | HIC | 2015 | 2005 | USA | Virological suppression | 12 | 1 month | Adults | 651 | Self report | No | Yes | Yes |
| Safren^96^ | SEARO | LMIC | 2005 |  | India | No missed doses | 58 | . | Adults | 75 | Self report | No | No | No |
| Saha^97^ | SEARO | LMIC | 2014 | 2011 | India | No missed doses | 52 | Ever | Adults | 201 | Self report | No | Yes | Yes |
| Seth^98^ | SEARO | LMIC | 2014 | 2011 | India | 95% | 108 | Ever | Children | 40 | Self report | No | Yes | No |
| Shumba^99^ | AFRO | LMIC | 2013 | 2009 | Uganda | No missed doses | 143 | 7 days | Adults | 60 | Self report | No | Yes | Yes |
| Simoni^100^ | AMRO N | HIC | 2002 | 1999 | USA | No missed doses | NR | 3 days | Adults | 50 | Self report | Yes | Yes | Yes |
| Sodergard^101^ | EURO | HIC | 2006 | 2004 | Sweden | NR | 146 |  | Adults | 239 | Self report | Yes | Yes | Yes |
| Spire^102^ | EURO | HIC | 2002 | 1998 | France | No missed doses | 17 | 4 days | Adults | 119 | Self report | No | Yes | No |
| Suleiman^103^ | AFRO | LMIC | 2016 | 2014 | Nigeria | No missed doses | 104 | Ever | Adults | 412 | Self report | No | Yes | Yes |
| Sullivan^104^ | AMRO N | HIC | 2007 | 2004 | USA | >95% | NS | 2 days | Adults | 821 | Self report | No | Yes | No |
| Tabatabai^105^ | AFRO | LMIC | 2014 | 2009 | Malawi | No missed appointment | NR | >21 days | Adults | 147 | Self report | No | Yes | No |
| Tadios^106^ | AFRO | LMIC | 2006 |  | Ethiopia | >95% | NR | 7 days | Adults | 81 | Self report | No | Yes | Yes |
| Talam^107^ | AFRO | LMIC | 2008 | 2005 | Kenya | >100% | NR | . | Adults | 218 | Self report | Yes | No | No |
| Tessema^108^ | AFRO | LMIC | 2010 | 2008 | Ethiopia | No missed doses | 67 | Ever | Adults | 87 | Self report | No | Yes | No |
| Tran^109^ | WPRO | LMIC | 2013 | 2012 | Vietnam | No missed doses | 158 | 1 month | Adults | 200 | Self report | No | Yes | No |
| Tsega^110^ | AFRO | LMIC | 2015 | 2014 | Ethiopia | No missed doses | 156 | 1 month | Adults | 67 | Self report | No | Yes | Yes |
| Ugwu^111^ | AFRO | LMIC | 2013 | 2011 | Nigeria | >95% | 152 | 1 month | Children | 87 | Self report | Yes | Yes | Yes |
| Uhagaze^112^ | AFRO | LMIC | 2006 | 2005 | Senegal | . | NR | . | Adults | 42 | Self report | No | No | No |
| Unge^113^ | AFRO | LMIC | 2010 | 2010 | Kenya | No missed doses | 100 | 1 month | Adults | 130 | Self report | No | Yes | Yes |
| Uzochukwu^114^ | AFRO | LMIC | 2009 |  | Nigeria | . | 151 | 1 month | Adults | 131 | Self report | Yes | Yes | No |
| Vallabhaneni^115^ | SEARO | LMIC | 2012 | 2009 | India | No missed doses | 78 | Ever | Adults | 110 | Self report | No | Yes | No |
| Vreeman^116^ | AFRO | LMIC | 2013 |  | Kenya | >90% | NR | 1 month | Children | 111 | Self report, MEMS | No | No | No |
| Wakibi^117^ | AFRO | LMIC | 2011 | 2009 | Kenya | >95% | 26-52 (median) | Ever | Adults | 50 | Self report | No | No | Yes |
| Walsh^118^ | EURO | HIC | 2001 | 1998 | UK | No missed doses | 114 | Ever | Adults | 157 | Self report | No | Yes | Yes |
| Wanchu^119^ | SEARO | LMIC | 2007 | 2005 | India | No missed doses | 59 | 1 month | Adults | 53 | Self report | No | No | Yes |
| Wang^120^ | WPRO | LMIC | 2007 | 2003 | China | 95% | . | Ever | Adults | 98 | Self report | No | Yes | Yes |
| Wang^121^ | WPRO | LMIC | 2008 | 2006 | China | No missed doses | 32 | 7 days | Adults | 64 | Self report | No | Yes | Yes |
| Wasti^122^ | SEARO | LMIC | 2012 | 2009 | Nepal | NR | 96 | 28 days | Adults | 48 | Self report | Yes | Yes | Yes |
| Weaver^123^ | WPRO | LMIC | 2014 | 2012 | Indonesia | <3 doses missed | 198 | 90 days | Adults | 57 | Self report | Yes | Yes | No |
| White^124^ | AMRO S | LMIC | 2008 | 2005 | Jamaica | No missed doses | 80 | 4 days | Children | 37 | Self report | No | Yes | Yes |
| van Dyke^125^ | AMRO N | HIC | 2002 | 1998 | USA | No missed doses | 48 | 3 days | Children | 46 | Self report | No | Yes | Yes |

AFRO=African region. AMRO N=region of the Americas (North). AMRO S=region of the Americas (South and Central). EURO=European region. EMRO=eastern Mediterranean region. HIC=high-income country. LMIC=low- and middle-income country. NR=not reported. SEARO=southeast Asia region. WPRO=western Pacific region. *No = No or not reported

References

1. Achappa B, Madi D, Bhaskaran U, Ramapuram JT, Rao S, Mahalingam S. Adherence to Antiretroviral Therapy Among People Living with HIV. *N Am J Med Sci* 2013; **5**(3): 220-3.

2. Aderemi-Williams R, Ajagbe L. Assessment of caregivers’ adherence to ARV drugs administration in HIV-Infected children. . *7th International Conference on HIV treatment and prevention adherence Miami, June 3-5 2012*; **Abstract 80009.**

3. Alakija Kazeem S, Fadeyi A, Ogunmodede JA, Desalu O. Factors influencing adherence to antiretroviral medication in Ilorin, Nigeria. *J Int Assoc Physicians AIDS Care (Chic)* 2010; **9**(3): 191-5.

4. Amberbir A, Woldemichael K, Getachew S, Girma B, Deribe K. Predictors of adherence to antiretroviral therapy among HIV-infected persons: a prospective study in Southwest Ethiopia. *BMC Public Health* 2008; **8**: 265.

5. Amico KR, Fisher WA, Cornman DH, et al. Visual analog scale of ART adherence: association with 3-day self-report and adherence barriers. *J Acquir Immune Defic Syndr* 2006; **42**(4): 455-9.

6. Ammassari A, Murri R, Pezzotti P, et al. Self-reported symptoms and medication side effects influence adherence to highly active antiretroviral therapy in persons with HIV infection. *J Acquir Immune Defic Syndr* 2001; **28**(5): 445-9.

7. Angevine R. Common HAART adherence barriers for adolescents. *Presentation at University of Virginia Dept of Public Health Sciences* 2007.

8. Arage G, Tessema GA, Kassa H. Adherence to antiretroviral therapy and its associated factors among children at South Wollo Zone Hospitals, Northeast Ethiopia: a cross-sectional study. *BMC Public Health* 2014; **14**: 365.

9. Arrondo Velasco A, Sainz Suberviola ML, Andres Esteban EM, Iruin Sanz AI, Napal Lecumberri V. [Factors associated with adherence in HIV patients]. *Farm Hosp* 2009; **33**(1): 4-11.

10. Babatunde O, Anyaike C, Elegbede O, Ayodele M, Abidoye S. Adherenceto HIV/AIDS antiretroviral therapy in a tertiary hospital In NorthCentral Nigeria. Abstract 48. *8th International Conference on HIV treatment and prevention adherence Miami, 2nd-4th June* 2013.

11. Baltazary G, Akarro RR, Mussa AS. Some factors associated with non-adherence to antiretroviral therapy (ART) in people living with HIV/AIDS (PLHA) in Tanzania: a case study of Dar es Salaam region. *East Afr J Public Health* 2011; **8**(4): 237-46.

12. Bant D. Cultural, ethnic, and socioeconomic issues in treatment initiation and adherence. Abstract 223. *8th International Conference on HIV treatment and prevention adherence Miami, 2nd-4th June* 2013.

13. Barfod TS, Sorensen HT, Nielsen H, Rodkjaer L, Obel N. 'Simply forgot' is the most frequently stated reason for missed doses of HAART irrespective of degree of adherence. *HIV Med* 2006; **7**(5): 285-90.

14. Belayneh M. Level of adherence to antiretroviral therapy and its determinants among people living with HIV AIDS in SNNP Region, Ethiopia. Abstract PUB017. *8th IAS Conference on HIV pathogenesis, treatment & prevention Vencouver, 19-22 July 2015*

15. Bello S. HIV/AIDS Patients’ Adherence To Antiretroviral Therapy In Sobi Specialist Hospital, Ilorin, Nigeria. *Global Journal of Medical research 11:2 16-25* 2011.

16. Bhat VG, Ramburuth M, Singh M, et al. Factors associated with poor adherence to anti-retroviral therapy in patients attending a rural health centre in South Africa. *Eur J Clin Microbiol Infect Dis* 2010; **29**(8): 947-53.

17. Biadgilign S, Deribew A, Amberbir A, Deribe K. Adherence to highly active antiretroviral therapy and its correlates among HIV infected pediatric patients in Ethiopia. *BMC Pediatr* 2008; **8**: 53.

18. Biressaw S, Abegaz WE, Abebe M, Taye WA, Belay M. Adherence to Antiretroviral Therapy and associated factors among HIV infected children in Ethiopia: unannounced home-based pill count versus caregivers' report. *BMC Pediatr* 2013; **13**: 132.

19. Blanco JM, Perez IR, De Labry Lima AO, Recio JM, Lopez EG, Basanta JJ. Adherence to antiretroviral treatment in prisons. *AIDS Res Hum Retroviruses* 2005; **21**(8): 683-8.

20. Buchanan AL, Montepiedra G, Sirois PA, et al. Barriers to medication adherence in HIV-infected children and youth based on self- and caregiver report. *Pediatrics* 2012; **129**(5): e1244-51.

21. Byakika-Tusiime J, Oyugi JH, Tumwikirize WA, Katabira ET, Mugyenyi PN, Bangsberg DR. Adherence to HIV antiretroviral therapy in HIV+ Ugandan patients purchasing therapy. *Int J STD AIDS* 2005; **16**(1): 38-41.

22. Chesney MA, Ickovics JR, Chambers DB, et al. Self-reported adherence to antiretroviral medications among participants in HIV clinical trials: the AACTG adherence instruments. Patient Care Committee & Adherence Working Group of the Outcomes Committee of the Adult AIDS Clinical Trials Group (AACTG). *AIDS Care* 2000; **12**(3): 255-66.

23. Cook RL, Sereika SM, Hunt SC, Woodward WC, Erlen JA, Conigliaro J. Problem drinking and medication adherence among persons with HIV infection. *J Gen Intern Med* 2001; **16**(2): 83-8.

24. Crozatti MT, Franca-Junior I, Rodrigues R, et al. Antiretroviral treatment adherence in childhood and adolescence: multidisciplinary team as an associated factor in Brazil. *AIDS Care* 2013; **25**(11): 1462-9.

25. Cummins D, Trotter G, Millar KH. Non-adherence to HIV antiretroviral medications: 'The drugs are working and I'm a continuing success story'. *Aust J Adv Nurs* 2002; **20**(2): 15-8.

26. Dachew BA, Tesfahunegn TB, Birhanu AM. Adherence to highly active antiretroviral therapy and associated factors among children at the University of Gondar Hospital and Gondar Poly Clinic, Northwest Ethiopia: a cross-sectional institutional based study. *BMC Public Health* 2014; **14**: 875.

27. Dashe A, Elimimian M, Egieyeh S, et al. Reasons for non-adherence amongst patients on HAART and the role of socioeconomic status,

educational level, gender and marital status. Abstract CDB361. *4th IAS conference on HIV pathogenesis, treatment and prevention Sydney 22-25 July 2007* 2007.

28. Davies MA, Boulle A, Fakir T, Nuttall J, Eley B. Adherence to antiretroviral therapy in young children in Cape Town, South Africa, measured by medication return and caregiver self-report: a prospective cohort study. *BMC Pediatr* 2008; **8**: 34.

29. DeMayo M, McFarlane A, Humphries K, Whyte K, Carty K. Adherence to HAART among people living with HIV/AIDS in the Atlanta EMA. Abstact MOPE508. *XIX International AIDS conference Washington, July 22-27th*.

30. Do H. Antiretroviral therapy (ART) adherence among people living with HIV/AIDS (PLHIV_ in the North of Vietnam: a multi-method approach. *Thesis submitted for the degree of Doctor of Philosophy at the School of Public Health, Faculty of Health, Queensland University of Technology* 2011.

31. Dyrehave C, Rasmussen DN, Honge BL, et al. Nonadherence is Associated with Lack of HIV-Related Knowledge: A Cross-Sectional Study among HIV-Infected Individuals in Guinea-Bissau. *J Int Assoc Provid AIDS Care* 2015.

32. Egieyeh S, Oqua D, Obodozie O, Kunle O, Dashe A, Inyang U. Reported reasons and demographic determinants of non adherence amongst clients on antiretroviral therapy in Federal Capital Territory of Nigeria. Abstract CDB0507. *XVII International AIDS Conference, Mexico City August 3-8th*

33. Ehlers VJ, Tshisuyi ET. Adherence to antiretroviral treatment by adults in a rural area of Botswana. *Curationis* 2015; **38**(1).

34. Eholie SP, Tanon A, Polneau S, et al. Field adherence to highly active antiretroviral therapy in HIV-infected adults in Abidjan, Cote d'Ivoire. *J Acquir Immune Defic Syndr* 2007; **45**(3): 355-8.

35. Ejekam C.S., Onajole A.T., Okany C.C., Lesi O.A., E.S. O. Adherence to antiretroviral drugs and its determinants among human immunodeficiency virus(HIV) patients attending HIV clinic in a teaching hospital in Nigeria. *Pharmacoepidemiology and Drug Safety Conference: 30th International Conference on Pharmacoepidemiology and Therapeutic Risk Management, Taipei 2014*.

36. Elise A, France AM, Louise WM, et al. Assessment of adherence to highly active antiretroviral therapy in a cohort of African HIV-infected children in Abidjan, Cote d'Ivoire. *J Acquir Immune Defic Syndr* 2005; **40**(4): 498-500.

37. Essomba EN, Adiogo D, Koum DC, Amang B, Lehman LG, Coppieters Y. [Factors associated with non-adherence of adults infected with HIV on antiretroviral therapy in a referral hospital in Douala]. *Pan Afr Med J* 2015; **20**: 412.

38. Eticha T, Berhane L. Caregiver-reported adherence to antiretroviral therapy among HIV infected children in Mekelle, Ethiopia. *BMC Pediatr* 2014; **14**: 114.

39. Fredericksen R, Church A, Harrington A, Harrington R. Identification of intentional non-adherence to HAART in routine care. Abstract 188. *8th International Conference on HIV treatment and prevention adherence Miami, 2nd-4th June* Dhanireddy, S.

Kitahata, M.

40. Gare J, Kelly-Hanku A, Ryan CE, et al. Factors Influencing Antiretroviral Adherence and Virological Outcomes in People Living with HIV in the Highlands of Papua New Guinea. *PLoS One* 2015; **10**(8): e0134918.

41. Genberg BL, Lee Y, Rogers WH, Wilson IB. Four types of barriers to adherence of antiretroviral therapy are associated with decreased adherence over time. *AIDS Behav* 2015; **19**(1): 85-92.

42. Gibb DM, Goodall RL, Giacomet V, et al. Adherence to prescribed antiretroviral therapy in human immunodeficiency virus-infected children in the PENTA 5 trial. *Pediatr Infect Dis J* 2003; **22**(1): 56-62.

43. Gifford AL, Bormann JE, Shively MJ, Wright BC, Richman DD, Bozzette SA. Predictors of self-reported adherence and plasma HIV concentrations in patients on multidrug antiretroviral regimens. *J Acquir Immune Defic Syndr* 2000; **23**(5): 386-95.

44. Gir E, Vaichulonis CG, de Oliveira MD. [Adhesion to anti-retroviral therapy by individuals with HIV/AIDS seen at an institution in the interior of Sao Paulo]. *Rev Lat Am Enfermagem* 2005; **13**(5): 634-41.

45. Gokarn A, Narkhede MG, Pardeshi GS, Doibale MK. Adherence to antiretroviral therapy. *J Assoc Physicians India* 2012; **60**: 16-20.

46. Golin CE, Liu H, Hays RD, et al. A prospective study of predictors of adherence to combination antiretroviral medication. *J Gen Intern Med* 2002; **17**(10): 756-65.

47. Guimaraes MD, Rocha GM, Campos LN, et al. Difficulties reported by HIV-infected patients using antiretroviral therapy in Brazil. *Clinics (Sao Paulo)* 2008; **63**(2): 165-72.

48. Hansana V, Sanchaisuriya P, Durham J, et al. Adherence to antiretroviral therapy (ART) among people living with HIV (PLHIV): a cross-sectional survey to measure in Lao PDR. *BMC Public Health* 2013; **13**: 617.

49. Haberer J, Kiwanuka J, Muzoora C, et al. Real-time HIV antiretroviral therapy adherence monitoring among adults and children in rural Uganda. Abstract 80027. *7th International Conference on HIV treatment and prevention adherence Miami, June 3-5 2012*.

50. Harris J, Pillinger M, Fromstein D, et al. Risk factors for medication non-adherence in an HIV infected population in the Dominican Republic. *AIDS Behav* 2011; **15**(7): 1410-5.

51. Hardon A, Davey S, Gerrits T, et al. From access to adherence: the challenges of antiretroviral treatment. Studies from Botswana, Tanzania and Uganda. *World Health Organization, Geneva* 2006.

52. Harvey KM, Carrington D, Duncan J, et al. Evaluation of adherence to highly active antiretroviral therapy in adults in Jamaica. *West Indian Med J* 2008; **57**(3): 293-7.

53. Heckman BD, Catz SL, Heckman TG, Miller JG, Kalichman SC. Adherence to antiretroviral therapy in rural persons living with HIV disease in the United States. *AIDS Care* 2004; **16**(2): 219-30.

54. Igwegbe A. O. , Ugboaja J. O., Nwajiaku L. A. Prevalence and determinants of non-adherence to antiretroviral therapy among HIV- positive pregnant women in Nnewi, Nigeria. *International Journal of Medicine and Medical Sciences 2: 238-245* 2010.

55. Iliyasu Z, Kabir M, Abubakar IS, Babashani M, Zubair ZA. Compliance to antiretroviral therapy among AIDS patients in Aminu Kano Teaching Hospital, Kano, Nigeria. *Niger J Med* 2005; **14**(3): 290-4.

56. Iroha E, Esezobor CI, Ezeaka C, Temiye EO, Akinsulie A. Adherence to antiretroviral therapy among HIV-infected children attending a donor-funded clinic at a tertiary hospital in Nigeria. *Afr J AIDS Res* 2010; **9**(1): 25-30.

57. Jean-Baptiste R. Factors associated with adherence to antiretroviral therapy in Rwanda. USAID. 2008.

58. Joshi K, Jhanwar S, Mathur A, Agarwal H, Mathur S. Barriers in adherence of ART (anti retroviral treatment): a experience of ART Centre of Western Rajasthan, India. Abstract CDB0504. *XVII International AIDS Conference, Mexico City August 3-8th*.

59. Kalichman SC, Catz S, Ramachandran B. Barriers to HIV/AIDS treatment and treatment adherence among African-American adults with disadvantaged education. *J Natl Med Assoc* 1999; **91**(8): 439-46.

60. Kerr T, Palepu A, Barness G, et al. Psychosocial determinants of adherence to highly active antiretroviral therapy among injection drug users in Vancouver. *Antivir Ther* 2004; **9**(3): 407-14.

61. Kleeberger CA, Phair JP, Strathdee SA, Detels R, Kingsley L, Jacobson LP. Determinants of heterogeneous adherence to HIV-antiretroviral therapies in the Multicenter AIDS Cohort Study. *J Acquir Immune Defic Syndr* 2001; **26**(1): 82-92.

62. Koole O, Denison JA, Menten J, et al. Reasons for Missing Antiretroviral Therapy: Results from a Multi-Country Study in Tanzania, Uganda, and Zambia. *PLoS One* 2016; **11**(1): e0147309.

63. Lal V, Kant S, Dewan R, Rai SK. Reasons for Non-adherence to Antiretroviral Therapy Among Adult Patients Receiving free Treatment at a Tertiary Care Hospital in Delhi. *Indian J Community Med* 2010; **35**(1): 172-3.

64. Letta S, Demissie A, Oljira L, Dessie Y. Factors associated with adherence to Antiretroviral Therapy (ART) among adult people living with HIV and attending their clinical care, Eastern Ethiopia. *BMC Int Health Hum Rights* 2015; **15**(1): 33.

65. MacDonell K, Naar-King S, Huszti H, Belzer M. Barriers to medication adherence in behaviorally and perinatally infected youth living with HIV. *AIDS Behav* 2013; **17**(1): 86-93.

66. Marhefka SL, Farley JJ, Rodrigue JR, Sandrik LL, Sleasman JW, Tepper VJ. Clinical assessment of medication adherence among HIV-infected children: examination of the Treatment Interview Protocol (TIP). *AIDS Care* 2004; **16**(3): 323-38.

67. Marhefka SL, Koenig LJ, Allison S, et al. Family experiences with pediatric antiretroviral therapy: responsibilities, barriers, and strategies for remembering medications. *AIDS Patient Care STDS* 2008; **22**(8): 637-47.

68. Marcos M, Worku A, Davey G. Adherence to ART in PLWHA at Yirgalem Hospital, South Ethiopia. *EthiopJHealth Dev* 2008; **22**: 174-9.

69. Mavhu W, Berwick J, Chirawu P, et al. Enhancing psychosocial support for HIV positive adolescents in Harare, Zimbabwe. *PLoS One* 2013; **8**(7): e70254.

70. Mbopi-Keou FX, Djomassi LD, Monebenimp F. [Descriptive aspects of HIV/AIDS in patients aged 50 years and over followed at the Treatment Center of Bafoussam - Cameroon]. *Pan Afr Med J* 2012; **12**: 107.

71. Mehta K, Ekstrand ML, Heylen E, Sanjeeva GN, Shet A. Adherence to Antiretroviral Therapy Among Children Living with HIV in South India. *AIDS Behav* 2015.

72. Moatti JP, Carrieri MP, Spire B, Gastaut JA, Cassuto JP, Moreau J. Adherence to HAART in French HIV-infected injecting drug users: the contribution of buprenorphine drug maintenance treatment. The Manif 2000 study group. *AIDS* 2000; **14**(2): 151-5.

73. Mohammed H, Kieltyka L, Richardson-Alston G, et al. Adherence to HAART among HIV-infected persons in rural Louisiana. *AIDS Patient Care STDS* 2004; **18**(5): 289-96.

74. Monreal MT, da Cunha RV, Trinca LA. Compliance to antiretroviral medication as reported by AIDS patients assisted at the University Hospital of the Federal University of Mato Grosso do Sul. *Braz J Infect Dis* 2002; **6**(1): 8-14.

75. Moralejo L, Ines S, Marcos M, Fuertes A, Luna G. Factors influencing adherence to highly active antiretroviral therapy in Spain. *Curr HIV Res* 2006; **4**(2): 221-7.

76. Morrison SD, Rashidi V, Sarnquist C, et al. Antiretroviral therapy adherence and predictors to adherence in Albania: a cross-sectional study. *J Infect Dev Ctries* 2014; **8**(7): 853-62.

77. Murphy DA, Sarr M, Durako SJ, et al. Barriers to HAART adherence among human immunodeficiency virus-infected adolescents. *Arch Pediatr Adolesc Med* 2003; **157**(3): 249-55.

78. Naik E, Casanas B, Pazare A, Wabale G, Sinnott J, Salihu H. Cost of treatment: The single biggest obstacle to HIV/AIDS treatment adherence in lower-middle class patients in Mumbai, India. *Indian J Sex Transm Dis* 2009; **30**(1): 23-7.

79. Nieuwkerk PT, Sprangers MA, Burger DM, et al. Limited patient adherence to highly active antiretroviral therapy for HIV-1 infection in an observational cohort study. *Arch Intern Med* 2001; **161**(16): 1962-8.

80. Nwauche c, Erhabor O, Ejele O, Akani C. Adherence to antiretroviral therapy among HIV-infected subjects in a resource - limited setting in the Niger Delta of Nigeria. *Afr J Health Sci* 2006; **2006**(13:13-17).

81. Nwokiki J. Baseline data and predictors of adherence in patients on antiretroviral therapy in Maun general hospital [MGH], Maun, Botswana. *Presentation* 2003.

82. Nyaku M, Beer L, Stockwell S. Non-persistence to antiretroviral therapy and viral suppression among HIV-infected adults in the United States. Abstract 22. *10th International conference on HIV treatment and prevention adherence June 28-30th, Miami* 2015.

83. Nyogea D, Geubbels E, Mtenga S, et al. Adherence patterns in HIV-positive patients in Southern rural Tanzania: The KIULARCO Cohort. Abstract 354. 2014; **9th International conference on HIV treatment and prevention adherence. 8-10th June, Miami**.

84. Obirikorang C, Selleh PK, Abledu JK, Fofie CO. Predictors of Adherence to Antiretroviral Therapy among HIV/AIDS Patients in the Upper West Region of Ghana. *ISRN AIDS* 2013; **2013**: 873939.

85. Odili VU, Obieche AO, Amibor KC. Adherence to Antiretroviral Therapy and Its Determinants Among HIV-Infected Patients in Nigeria. *J Pharm Pract* 2016.

86. Okonsky JG, Webel A, Rose CD, et al. Appreciating Reasons for Nonadherence in Women. *Health Care Women Int* 2015; **36**(9): 1007-25.

87. Okoronkwo I, Okeke U, Chinweuba A, Iheanacho P. Nonadherence Factors and Sociodemographic Characteristics of HIV-Infected Adults Receiving Antiretroviral Therapy in Nnamdi Azikiwe University Teaching Hospital, Nnewi, Nigeria. *ISRN AIDS* 2013; **2013**: 843794.

88. Oku AO, Owoaje ET, Ige OK, Oyo-Ita A. Prevalence and determinants of adherence to HAART amongst PLHIV in a tertiary health facility in south-south Nigeria. *BMC Infect Dis* 2013; **13**: 401.

89. Oku AO, Owoaje ET, Oku OO, Monjok E. Prevalence and determinants of adherence to highly active antiretroviral therapy amongst people living with HIV/AIDS in a rural setting in south-south Nigeria. *Afr J Reprod Health* 2014; **18**(1): 133-43.

90. Olowookere SA, Fatiregun AA, Akinyemi JO, Bamgboye AE, Osagbemi GK. Prevalence and determinants of nonadherence to highly active antiretroviral therapy among people living with HIV/AIDS in Ibadan, Nigeria. *J Infect Dev Ctries* 2008; **2**(5): 369-72.

91. Pennap G, Abdullahi U, Bako I. Adherence to highly active antiretroviral therapy and its challenges in people living with human immunodeficiency virus (HIV) infection in Keffi, Nigeria. *Journal of AIDS and HIV Research* 2013; **5**: 52-8.

92. Rao D, Kekwaletswe TC, Hosek S, Martinez J, Rodriguez F. Stigma and social barriers to medication adherence with urban youth living with HIV. *AIDS Care* 2007; **19**(1): 28-33.

93. Remien RH, Bastos FI, Jnr VT, et al. Adherence to antiretroviral therapy in a context of universal access, in Rio de Janeiro, Brazil. *AIDS Care* 2007; **19**(6): 740-8.

94. Riera M, La Fuente Ld L, Castanyer B, et al. [Adherence to antiretroviral therapy measured by pill count and drug serum concentrations. Variables associated with a bad adherence]. *Med Clin (Barc)* 2002; **119**(8): 286-92.

95. Saberi P, Neilands TB, Vittinghoff E, Johnson MO, Chesney M, Cohn SE. Barriers to antiretroviral therapy adherence and plasma HIV RNA suppression among AIDS clinical trials group study participants. *AIDS Patient Care STDS* 2015; **29**(3): 111-6.

96. Safren SA, Kumarasamy N, James R, Raminani S, Solomon S, Mayer KH. ART adherence, demographic variables and CD4 outcome among HIV-positive patients on antiretroviral therapy in Chennai, India. *AIDS Care* 2005; **17**(7): 853-62.

97. Saha R, Saha I, Sarkar AP, et al. Adherence to highly active antiretroviral therapy in a tertiary care hospital in West Bengal, India. *Singapore Med J* 2014; **55**(2): 92-8.

98. Seth A, Gupta R, Chandra J, Maheshwari A, Kumar P, Aneja S. Adherence to antiretroviral therapy and its determinants in children with HIV infection - Experience from Paediatric Centre of Excellence in HIV Care in North India. *AIDS Care* 2014; **26**(7): 865-71.

99. Shumba C, Atuhaire L, Imakit R, Atukunda R, Memiah P. Missed Doses and Missed Appointments: Adherence to ART among Adult Patients in Uganda. *ISRN AIDS* 2013; **2013**: 270914.

100. Simoni JM, Frick PA, Lockhart D, Liebovitz D. Mediators of social support and antiretroviral adherence among an indigent population in New York City. *AIDS Patient Care STDS* 2002; **16**(9): 431-9.

101. Sodergard B, Halvarsson M, Tully MP, et al. Adherence to treatment in Swedish HIV-infected patients. *J Clin Pharm Ther* 2006; **31**(6): 605-16.

102. Spire B, Duran S, Souville M, et al. Adherence to highly active antiretroviral therapies (HAART) in HIV-infected patients: from a predictive to a dynamic approach. *Soc Sci Med* 2002; **54**(10): 1481-96.

103. Suleiman IA, Momo A. Adherence to antiretroviral therapy and its determinants among persons living with HIV/AIDS in Bayelsa state, Nigeria. *Pharm Pract (Granada)* 2016; **14**(1): 631.

104. Sullivan PS, Campsmith ML, Nakamura GV, Begley EB, Schulden J, Nakashima AK. Patient and regimen characteristics associated with self-reported nonadherence to antiretroviral therapy. *PLoS One* 2007; **2**(6): e552.

105. Tabatabai J, Namakhoma I, Tweya H, Phiri S, Schnitzler P, Neuhann F. Understanding reasons for treatment interruption amongst patients on antiretroviral therapy--a qualitative study at the Lighthouse Clinic, Lilongwe, Malawi. *Glob Health Action* 2014; **7**: 24795.

106. Tadios Y, Davey G. Antiretroviral treatment adherence and its correlates in Addis Ababa, Ethiopia. *Ethiop Med J* 2006; **44**(3): 237-44.

107. Talam NC, Gatongi P, Rotich J, Kimaiyo S. Factors affecting antiretroviral drug adherence among HIV/AIDS adult patients attending HIV/AIDS clinic at Moi Teaching and Referral Hospital, Eldoret, Kenya. *East Afr J Public Health* 2008; **5**(2): 74-8.

108. Tessema B, Biadglegne F, Mulu A, Getachew A, Emmrich F, Sack U. Magnitude and determinants of nonadherence and nonreadiness to highly active antiretroviral therapy among people living with HIV/AIDS in Northwest Ethiopia: a cross-sectional study. *AIDS Res Ther* 2010; **7**: 2.

109. Tran BX, Nguyen LT, Nguyen NH, Hoang QV, Hwang J. Determinants of antiretroviral treatment adherence among HIV/AIDS patients: a multisite study. *Glob Health Action* 2013; **6**: 19570.

110. Tsega B, Srikanth BA, Shewamene Z. Determinants of non-adherence to antiretroviral therapy in adult hospitalized patients, Northwest Ethiopia. *Patient Prefer Adherence* 2015; **9**: 373-80.

111. Ugwu R, Eneh A. Factors influencing adherence to paediatric antiretroviral therapy in Portharcourt, South- South Nigeria. *Pan Afr Med J* 2013; **16**: 30.

112. Uhagaze B, Ndour T, Sow P, Rahlenbeck S. Why are drugs not taken? a study into ARV-therapy interruptions in Dakar, Senegal. Abstract CDB0813. **XVI International AIDS Conference, Toronto 13-18th August, 2006**.

113. Unge C, Sodergard B, Marrone G, et al. Long-term adherence to antiretroviral treatment and program drop-out in a high-risk urban setting in sub-Saharan Africa: a prospective cohort study. *PLoS One* 2010; **5**(10): e13613.

114. Uzochukwu BS, Onwujekwe OE, Onoka AC, Okoli C, Uguru NP, Chukwuogo OI. Determinants of non-adherence to subsidized anti-retroviral treatment in southeast Nigeria. *Health Policy Plan* 2009; **24**(3): 189-96.

115. Vallabhaneni S, Chandy S, Heylen E, Ekstrand M. Reasons for and correlates of antiretroviral treatment interruptions in a cohort of patients from public and private clinics in southern India. *AIDS Care* 2012; **24**(6): 687-94.

116. Vreeman R, Nyandiko W, Liu H, et al. Antiretroviral therapy adherence patterns among HIV-infected Kenyan children. Abstract WEPE527. *7th IAS Conference on HIV pathogenesis, treatment, and prevention Kuala Lumpur June 30th - 3rd July, 2013*.

117. Wakibi SN, Ng'ang'a ZW, Mbugua GG. Factors associated with non-adherence to highly active antiretroviral therapy in Nairobi, Kenya. *AIDS Res Ther* 2011; **8**: 43.

118. Walsh JC, Horne R, Dalton M, Burgess AP, Gazzard BG. Reasons for non-adherence to antiretroviral therapy: patients' perspectives provide evidence of multiple causes. *AIDS Care* 2001; **13**(6): 709-20.

119. Wanchu A, Kaur R, Bambery P, Singh S. Adherence to generic reverse transcriptase inhibitor-based antiretroviral medication at a Tertiary Center in North India. *AIDS Behav* 2007; **11**(1): 99-102.

120. Wang X, Wu Z. Factors associated with adherence to antiretroviral therapy among HIV/AIDS patients in rural China. *AIDS* 2007; **21 Suppl 8**: S149-55.

121. Wang H, He G, Li X, et al. Self-Reported adherence to antiretroviral treatment among HIV-infected people in Central China. *AIDS Patient Care STDS* 2008; **22**(1): 71-80.

122. Wasti SP, Simkhada P, Randall J, Freeman JV, van Teijlingen E. Factors influencing adherence to antiretroviral treatment in Nepal: a mixed-methods study. *PLoS One* 2012; **7**(5): e35547.

123. Weaver ER, Pane M, Wandra T, Windiyaningsih C, Herlina, Samaan G. Factors that influence adherence to antiretroviral treatment in an urban population, Jakarta, Indonesia. *PLoS One* 2014; **9**(9): e107543.

124. White YR, Pierre RB, Steel-Duncan J, et al. Adherence to antiretroviral drug therapy in children with HIV/AIDS in Jamaica. *West Indian Med J* 2008; **57**(3): 231-7.

125. Van Dyke RB, Lee S, Johnson GM, et al. Reported adherence as a determinant of response to highly active antiretroviral therapy in children who have human immunodeficiency virus infection. *Pediatrics* 2002; **109**(4): e61.
