# Supplementary material for: Patient-Reported Barriers to Adherence to Antiretroviral Therapy: A Systematic Review and Meta-Analysis
Source: PLoS Med. 2016 Nov 29;13(11):e1002183. doi: 10.1371/journal.pmed.1002183 (PMC5127502; doi:10.1371/journal.pmed.1002183)
Supplement: S2 Table — (DOCX) [file pmed.1002183.s002.docx]

**Table S2: Patient reported barriers to adherence**

| **Content** | **Pages** |
| --- | --- |
| Patient report barriers to adherence: Adults | 2-31 |
| Patient report barriers to adherence: Children | 32-45 |
| Patient report barriers to adherence: Adolescents | 46-61 |
|  |  |

**Patient report barriers to adherence: Adults**

**Forgot**

Study | % [95% Conf. Interval]

---------------------+-----------------------------------------------

SEARO

Achappa | 30.846 18.599 44.648

Bant | 44.505 34.482 54.760

Gokarn | 14.363 7.831 22.462

Lal | 31.964 20.949 44.110

Saha | 70.050 63.563 76.156

Wanchu | 23.142 12.976 35.207

Wasti | 35.711 23.026 49.509

Sub-total |

D+L pooled % | 35.913 17.789 54.038

---------------------+-----------------------------------------------

AFRO

Amberbir | 43.750 33.596 54.175

Babatunde | 42.681 28.116 57.925

Baltazary | 12.373 8.166 17.309

Belayneh | 8.527 4.812 13.177

Bello | 5.529 1.291 12.464

Bhat | 41.405 29.715 53.609

Byakika-Tusiime | 19.628 13.391 26.728

Dyrehave | 22.929 19.336 26.733

Ehlers | 33.618 22.134 46.181

Ejekam | 51.005 44.072 57.919

Essomba | 32.752 27.168 38.592

Haberer | 9.987 3.946 18.381

Hardon | 44.802 38.015 51.687

Igwebe | 63.581 52.864 73.657

Ilyaso | 23.891 18.290 29.985

Jean-Baptiste | 30.182 23.416 37.412

Koole | 53.284 50.546 56.012

Letta | 39.893 30.267 49.931

Markos | 50.658 39.496 61.787

Mbopi-Kéou | 22.701 16.800 29.203

Nwokike | 65.133 54.128 75.376

Nyogea | 48.082 42.976 53.209

Obirikorang | 46.104 35.159 57.242

Odili | 72.592 67.419 77.476

Okoronkwo | 53.704 46.581 60.751

Oku | 31.024 24.233 38.253

Oku | 48.724 41.762 55.712

Olowookere | 55.530 48.889 62.073

Pennap | 51.238 44.355 58.097

Shumba | 28.685 18.121 40.584

Suleiman | 35.714 31.167 40.395

Tabatabai | 20.607 14.499 27.476

Tadios | 33.535 23.780 44.058

Talam | 50.913 44.304 57.507

Tsega | 43.382 31.899 55.237

Uhagaze | 26.736 14.735 40.800

Unge | 77.482 69.962 84.198

Sub-total |

D+L pooled % | 38.892 32.591 45.194

---------------------+-----------------------------------------------

AMRO N

Amico | 48.630 37.301 60.031

Chesney | 66.350 53.064 78.435

Cook | 39.679 33.294 46.245

DeMayo | 47.041 39.578 54.572

Fredericksen | 15.098 6.103 27.188

Genberg | 46.925 42.585 51.288

Heckman | 15.232 9.559 21.947

Kalichman | 47.183 35.751 58.766

Kerr | 27.063 19.174 35.758

Kleeberger | 74.795 66.706 82.097

Mohammed | 22.558 17.232 28.373

Okonsky | 70.110 60.402 78.981

Saberi | 45.015 41.215 48.845

Simoni | 50.000 36.449 63.551

Sullivan | 32.421 29.265 35.659

Sub-total |

D+L pooled % | 42.895 35.010 50.781

---------------------+-----------------------------------------------

EURO

Barfod | 83.986 77.156 89.799

Blanco | 43.548 35.855 51.401

Harris | 40.971 29.953 52.470

Moatti | 45.689 33.152 58.510

Morrison | 21.010 12.290 31.336

Nieuwkerk | 35.377 26.593 44.688

Riera | 32.497 21.330 44.780

Spire | 36.249 27.914 45.024

Walsh | 35.126 27.893 42.720

Sub-total |

D+L pooled % | 41.728 27.326 56.130

---------------------+-----------------------------------------------

AMRO S

Guimarães | 37.341 27.067 48.227

Harvey | 37.179 28.680 46.098

Monreal | 67.554 57.797 76.597

Remien | 54.975 48.072 61.784

Sub-total |

D+L pooled % | 49.383 35.743 63.024

---------------------+-----------------------------------------------

WPRO

Cummings | 81.587 70.579 90.478

Do | 32.549 28.907 36.299

Gare | 42.707 29.189 56.804

Hansana | 62.133 53.848 70.076

Tran | 21.641 16.237 27.587

Wang | 50.000 37.964 62.036

Wang | 47.980 38.241 57.796

Weaver | 66.382 53.819 77.866

Sub-total |

D+L pooled % | 50.361 36.438 64.283

---------------------+-----------------------------------------------

Overall |

D+L pooled % | 41.382 37.314 45.449

---------------------+-----------------------------------------------

**Travel**

Study | % [95% Conf. Interval]

---------------------+-----------------------------------------------

SEARO

Gokarn | 59.196 48.742 69.245

Lal | 35.244 23.843 47.569

Safren | 5.904 1.759 12.268

Saha | 65.099 58.403 71.508

Vallabhaneni | 12.159 6.767 18.856

Wanchu | 12.022 4.793 21.936

Wasti | 27.545 16.074 40.765

Sub-total |

D+L pooled % | 30.932 11.324 50.541

---------------------+-----------------------------------------------

AFRO

Amberbir | 9.652 4.404 16.654

Babatunde | 45.120 30.352 60.342

Baltazary | 21.464 16.040 27.441

Belayneh | 12.057 7.609 17.362

Bello | 18.247 9.770 28.651

Bhat | 57.032 44.815 68.829

Byakika-Tusiime | 11.479 6.674 17.377

Dashe | 27.013 24.393 29.716

Dyrehave | 15.858 12.777 19.205

Egieyeh | 16.834 10.122 24.841

Ejekam | 23.366 17.763 29.483

Essomba | 8.332 5.278 12.006

Haberer | 2.243 0.083 7.212

Jean-Baptiste | 25.304 18.965 32.221

Koole | 2.775 1.947 3.746

Letta | 21.806 14.089 30.671

Markos | 9.859 4.223 17.515

Mbopi-Kéou | 8.906 5.142 13.575

Nwauche | 3.628 0.837 8.268

Nwokike | 49.342 38.213 60.504

Obirikorang | 16.229 8.900 25.235

Oku | 14.758 9.790 20.539

Oku | 15.050 10.403 20.382

Olowookere | 33.410 27.299 39.814

Pennap | 19.059 13.953 24.752

Shumba | 50.000 37.584 62.416

Tabatabai | 54.392 46.346 62.325

Tadios | 27.437 18.381 37.546

Talam | 71.005 64.829 76.813

Unge | 45.420 37.002 53.971

Sub-total |

D+L pooled % | 23.998 18.518 29.479

---------------------+-----------------------------------------------

AMRO N

Amico | 39.040 28.234 50.420

Chesney | 56.732 43.184 69.786

DeMayo | 14.496 9.611 20.186

Genberg | 34.028 29.957 38.220

Gifford | 46.323 34.686 58.167

Golin | 59.029 47.530 70.048

Kalichman | 23.236 14.221 33.692

Kerr | 17.888 11.299 25.604

Kleeberger | 59.918 51.075 68.446

Mohammed | 16.511 11.862 21.758

Okonsky | 57.066 46.874 66.963

Saberi | 50.383 46.548 54.216

Simoni | 38.233 25.503 51.844

Sub-total |

D+L pooled % | 39.078 29.587 48.568

---------------------+-----------------------------------------------

EURO

Ammassari | 35.624 25.557 46.378

Barfod | 77.736 70.149 84.492

Harris | 31.248 21.135 42.357

Morrison | 18.110 9.991 27.996

Spire | 17.915 11.602 25.252

Sub-total |

D+L pooled % | 36.176 11.198 61.154

---------------------+-----------------------------------------------

AMRO S

Harvey | 38.034 29.481 46.978

Remien | 48.010 41.144 54.914

Sub-total |

D+L pooled % | 43.402 33.654 53.150

---------------------+-----------------------------------------------

WPRO

Do | 40.990 37.139 44.898

Tran | 6.714 3.678 10.576

Wang | 56.155 44.029 67.918

Weaver | 37.067 25.200 49.786

Sub-total |

D+L pooled % | 34.890 11.397 58.383

---------------------+-----------------------------------------------

Overall |

D+L pooled % | 30.381 25.544 35.218

---------------------+-----------------------------------------------

**Busy**

Study | % [95% Conf. Interval]

---------------------+-----------------------------------------------

SEARO

Gokarn | 33.907 24.386 44.135

Lal | 5.710 1.335 12.859

Safren | 12.493 6.066 20.807

Saha | 64.604 57.892 71.039

Vallabhaneni | 4.042 1.192 8.478

Wasti | 50.000 36.182 63.818

Sub-total |

D+L pooled % | 28.193 7.325 49.061

---------------------+-----------------------------------------------

AFRO

Amberbir | 5.099 1.513 10.638

Baltazary | 25.505 19.690 31.794

Belayneh | 6.762 3.489 11.010

Bello | 7.122 2.136 14.710

Bhat | 49.219 37.116 61.368

Byakika-Tusiime | 6.291 2.835 10.988

Dashe | 38.998 36.094 41.941

Egieyeh | 26.019 17.858 35.117

Essomba | 12.984 9.168 17.350

Hardon | 30.445 24.305 36.957

Jean-Baptiste | 11.279 6.909 16.554

Letta | 10.100 4.863 16.963

Mbopi-Kéou | 19.827 14.257 26.061

Obirikorang | 4.523 1.050 10.256

Odili | 18.438 14.268 23.011

Okoronkwo | 38.889 32.075 45.928

Oku | 43.675 36.232 51.264

Oku | 50.765 43.782 57.734

Shumba | 4.059 0.598 10.398

Suleiman | 10.532 7.760 13.672

Unge | 20.228 13.818 27.508

Uzochukwu | 10.224 5.658 15.945

Sub-total |

D+L pooled % | 20.168 14.338 25.998

---------------------+-----------------------------------------------

AMRO N

Amico | 21.229 12.684 31.281

Chesney | 52.885 39.378 66.180

Genberg | 37.996 33.810 42.274

Kalichman | 52.817 41.234 64.249

Kerr | 21.558 14.383 29.732

Kleeberger | 69.836 61.387 77.656

Mohammed | 14.650 10.253 19.678

Okonsky | 59.240 49.076 69.019

Saberi | 39.187 35.476 42.962

Simoni | 38.233 25.503 51.844

Sullivan | 10.280 8.298 12.447

Sub-total |

D+L pooled % | 37.643 25.860 49.426

---------------------+-----------------------------------------------

EURO

Arrondo-Velasco | 17.665 12.001 24.156

Barfod | 69.141 60.893 76.817

Harris | 17.356 9.552 26.893

Moatti | 21.545 12.018 32.947

Morrison | 18.110 9.991 27.996

Nieuwkerk | 24.055 16.436 32.611

Riera | 10.819 4.291 19.843

Spire | 27.082 19.540 35.357

Walsh | 27.531 20.867 34.740

Sub-total |

D+L pooled % | 25.942 14.818 37.066

---------------------+-----------------------------------------------

AMRO S

Remien | 43.035 36.278 49.924

Sub-total |

D+L pooled % | 43.035 36.212 49.858

---------------------+-----------------------------------------------

WPRO

Do | 38.068 34.275 41.936

Gare | 28.119 16.439 41.538

Hansana | 96.701 93.055 99.031

Tran | 33.084 26.762 39.730

Wang | 48.461 36.476 60.537

Wang | 31.817 23.053 41.284

Weaver | 62.933 50.214 74.800

Sub-total |

D+L pooled % | 48.533 21.526 75.540

---------------------+-----------------------------------------------

Overall |

D+L pooled % | 29.383 23.066 35.700

---------------------+-----------------------------------------------

**Change to routine**

Study | % [95% Conf. Interval]

---------------------+-----------------------------------------------

SEARO

Lal | 7.356 2.209 15.174

Saha | 40.346 33.694 47.182

Sub-total |

D+L pooled % | 23.838 0.000 56.168

---------------------+-----------------------------------------------

AFRO

Amberbir | 3.958 0.915 9.003

Bhat | 24.214 14.594 35.375

Dashe | 37.968 35.081 40.899

Egieyeh | 21.937 14.345 30.624

Jean-Baptiste | 5.179 2.324 9.081

Markos | 11.176 5.129 19.177

Talam | 14.383 10.061 19.329

Sub-total |

D+L pooled % | 16.897 4.602 29.193

---------------------+-----------------------------------------------

AMRO N

Amico | 14.377 7.332 23.288

Chesney | 50.962 37.506 64.347

Genberg | 26.091 22.353 30.011

Gifford | 44.852 33.287 56.707

Golin | 42.360 31.251 53.876

Kerr | 13.299 7.607 20.282

Kleeberger | 74.795 66.706 82.097

Saberi | 44.862 41.063 48.691

Sub-total |

D+L pooled % | 38.706 25.373 52.039

---------------------+-----------------------------------------------

EURO

Barfod | 71.485 63.381 78.946

Harris | 10.406 4.468 18.448

Moatti | 21.545 12.018 32.947

Morrison | 13.760 6.711 22.817

Nieuwkerk | 19.337 12.418 27.365

Sub-total |

D+L pooled % | 27.315 4.512 50.117

---------------------+-----------------------------------------------

AMRO S

Remien | 35.074 28.641 41.793

Sub-total |

D+L pooled % | 35.074 28.498 41.651

---------------------+-----------------------------------------------

WPRO

Do | 34.172 30.480 37.963

Weaver | 19.821 10.668 30.961

Sub-total |

D+L pooled % | 27.803 13.828 41.778

---------------------+-----------------------------------------------

Overall |

D+L pooled % | 27.966 20.897 35.035

---------------------+-----------------------------------------------

**Asleep**

Study | % [95% Conf. Interval]

---------------------+-----------------------------------------------

SEARO

Lal | 4.059 0.598 10.398

Saha | 34.406 28.023 41.084

Wasti | 17.335 8.146 29.068

Sub-total |

D+L pooled % | 18.547 0.012 39.106

---------------------+-----------------------------------------------

AFRO

Amberbir | 6.238 2.173 12.206

Bhat | 30.466 19.885 42.213

Dashe | 19.054 16.756 21.464

Egieyeh | 15.813 9.304 23.658

Essomba | 4.843 2.566 7.792

Markos | 7.223 2.529 14.071

Mbopi-Kéou | 10.630 6.501 15.627

Olowookere | 26.958 21.275 33.049

Suleiman | 6.416 4.259 8.978

Uhagaze | 15.098 6.103 27.188

Unge | 5.719 2.410 10.324

Sub-total |

D+L pooled % | 12.784 8.175 17.393

---------------------+-----------------------------------------------

AMRO N

Amico | 25.339 16.095 35.876

Chesney | 39.421 26.690 52.929

Cook | 28.211 22.446 34.359

Genberg | 32.044 28.043 36.181

Golin | 38.193 27.384 49.630

Kalichman | 40.140 29.106 51.705

Kerr | 24.310 16.756 32.767

Kleeberger | 48.347 39.516 57.230

Mohammed | 18.372 13.493 23.815

Okonsky | 59.240 49.076 69.019

Saberi | 43.328 39.548 47.148

Sub-total |

D+L pooled % | 35.719 28.878 42.561

---------------------+-----------------------------------------------

EURO

Barfod | 53.516 44.866 62.060

Harris | 7.624 2.674 14.825

Morrison | 7.956 2.795 15.447

Spire | 22.915 15.868 30.827

Walsh | 36.392 29.086 44.029

Sub-total |

D+L pooled % | 25.513 9.183 41.842

---------------------+-----------------------------------------------

AMRO S

Harvey | 37.179 28.680 46.098

Remien | 34.577 28.170 41.278

Sub-total |

D+L pooled % | 35.518 30.281 40.755

---------------------+-----------------------------------------------

WPRO

Do | 34.010 30.322 37.797

Tran | 11.690 7.628 16.484

Wang | 5.545 1.926 10.883

Weaver | 59.484 46.677 71.667

Sub-total |

D+L pooled % | 26.855 9.368 44.342

---------------------+-----------------------------------------------

Overall |

D+L pooled % | 24.849 20.119 29.578

---------------------+-----------------------------------------------

**Avoid side effects**

Study | % [95% Conf. Interval]

---------------------+-----------------------------------------------

SEARO

Gokarn | 1.676 0.061 5.416

Lal | 5.710 1.335 12.859

Saha | 10.147 6.375 14.676

Wasti | 60.206 46.282 73.335

Sub-total |

D+L pooled % | 17.009 4.709 29.308

---------------------+-----------------------------------------------

AFRO

Eholie | 26.907 21.452 32.737

Essomba | 3.292 1.467 5.811

Markos | 1.918 0.070 6.186

Oku | 2.703 0.792 5.706

Oku | 3.822 1.599 6.950

Olowookere | 35.253 29.046 41.722

Pennap | 16.088 11.359 21.461

Suleiman | 8.111 5.677 10.934

Uhagaze | 70.937 56.639 83.379

Unge | 3.425 1.007 7.205

Sub-total |

D+L pooled % | 15.006 9.200 20.813

---------------------+-----------------------------------------------

AMRO N

Amico | 7.520 2.636 14.629

Chesney | 24.032 13.514 36.456

Fredericksen | 19.755 9.372 32.819

Genberg | 12.004 9.315 14.982

Gifford | 16.905 9.011 26.660

Heckman | 12.107 7.051 18.297

Kerr | 12.382 6.896 19.190

Kleeberger | 48.347 39.516 57.230

Mohammed | 14.185 9.855 19.155

Nyaku | 18.149 14.598 21.994

Okonsky | 45.108 35.113 55.308

Saberi | 20.475 17.468 23.657

Sub-total |

D+L pooled % | 20.058 15.057 25.059

---------------------+-----------------------------------------------

EURO

Ammassari | 41.874 31.344 52.793

Barfod | 25.390 18.256 33.260

Harris | 14.576 7.439 23.596

Morrison | 5.048 1.175 11.411

Spire | 13.747 8.198 20.454

Sub-total |

D+L pooled % | 19.535 8.735 30.336

---------------------+---------------------------------------------------

AMRO S

Monreal | 21.806 14.089 30.671

Remien | 24.626 18.931 30.806

Sub-total |

D+L pooled % | 23.670 18.843 28.498

---------------------+-----------------------------------------------

WPRO

Do | 32.549 28.907 36.299

Wang | 22.303 13.097 33.138

Weaver | 23.270 13.396 34.906

Sub-total |

D+L pooled % | 27.344 19.724 34.965

---------------------+-----------------------------------------------

Overall |

D+L pooled % | 19.068 15.373 22.763

---------------------+-----------------------------------------------

**Toxicity**

Study | % [95% Conf. Interval]

---------------------+-----------------------------------------------

SEARO

Achappa | 54.256 40.034 68.133

Bant | 17.029 10.054 25.392

Gokarn | 18.962 11.468 27.822

Joshi | 4.773 1.414 9.975

Lal | 2.390 0.089 7.675

Safren | 12.493 6.066 20.807

Vallabhaneni | 25.674 18.014 34.174

Wanchu | 10.166 3.610 19.540

Sub-total |

D+L pooled % | 16.897 8.899 24.894

---------------------+-----------------------------------------------

AFRO

Babatunde | 37.801 23.756 52.980

Baltazary | 53.283 46.324 60.178

Bello | 23.011 13.542 34.118

Bhat | 50.781 38.632 62.884

Byakika-Tusiime | 11.479 6.674 17.377

Dyrehave | 14.444 11.489 17.675

Igwebe | 5.539 1.647 11.532

Koole | 12.314 10.571 14.171

Letta | 9.036 4.114 15.626

Nwauche | 18.226 11.189 26.531

Nwokike | 37.499 27.021 48.606

Odili | 8.803 5.872 12.260

Okoronkwo | 32.010 25.564 38.821

Shumba | 2.390 0.089 7.675

Tabatabai | 3.031 0.889 6.390

Tessema | 31.249 22.043 41.270

Tsega | 3.641 0.534 9.354

Unge | 3.425 1.007 7.205

Uzochukwu | 76.137 68.522 82.994

Sub-total |

D+L pooled % | 22.073 15.753 28.393

---------------------+-----------------------------------------------

AMRO N

Chesney | 12.484 4.988 22.735

Cook | 17.201 12.495 22.485

DeMayo | 14.496 9.611 20.186

Genberg | 11.012 8.431 13.890

Gifford | 18.377 10.147 28.384

Golin | 29.859 19.917 40.871

Heckman | 12.107 7.051 18.297

Kalichman | 23.236 14.221 33.692

Kerr | 5.036 1.745 9.907

Kleeberger | 34.297 26.132 42.959

Mohammed | 10.929 7.120 15.436

Saberi | 12.653 10.214 15.313

Sullivan | 18.552 15.970 21.281

Sub-total |

D+L pooled % | 15.822 12.540 19.104

---------------------+-----------------------------------------------

EURO

Arrondo-Velasco | 38.333 30.735 46.230

Barfod | 16.014 10.201 22.844

Blanco | 22.257 16.079 29.122

Harris | 2.025 0.074 6.524

Moatti | 28.444 17.671 40.637

Moralejo | 12.285 7.006 18.788

Morrison | 7.956 2.795 15.447

Riera | 12.488 5.407 21.958

Sodergard | 24.375 19.162 29.997

Walsh | 20.569 14.649 27.203

Sub-total |

D+L pooled % | 18.177 10.989 25.365

---------------------+-----------------------------------------------

AMRO S

Gir | 34.713 27.483 42.316

Guimarães | 19.616 11.666 29.038

Harvey | 19.229 12.631 26.835

Remien | 13.183 8.872 18.196

Sub-total |

D+L pooled % | 21.492 12.076 30.907

---------------------+-----------------------------------------------

WPRO

Do | 27.354 23.908 30.941

Hansana | 4.773 1.844 8.976

Tran | 3.229 1.239 6.111

Wang | 27.776 19.440 36.972

Weaver | 18.095 9.347 28.945

Sub-total |

D+L pooled % | 15.953 4.448 27.458

---------------------+-----------------------------------------------

Overall |

D+L pooled % | 18.770 15.942 21.598

---------------------+-----------------------------------------------

**Ran out of pills**

Study | % [95% Conf. Interval]

---------------------+-----------------------------------------------

SEARO

Lal | 5.710 1.335 12.859

Safren | 13.809 7.029 22.411

Saha | 5.195 2.570 8.671

Wanchu | 15.730 7.345 26.537

Wasti | 64.289 50.491 76.974

Sub-total |

D+L pooled % | 19.602 6.649 32.555

---------------------+-----------------------------------------------

AFRO

Amberbir | 13.063 6.876 20.856

Babatunde | 32.921 19.554 47.876

Belayneh | 5.585 2.647 9.524

Bhat | 33.592 22.626 45.537

Dyrehave | 20.707 17.256 24.386

Ehlers | 21.545 12.018 32.947

Jean-Baptiste | 8.229 4.528 12.907

Letta | 9.036 4.114 15.626

Nyogea | 19.589 15.684 23.813

Obirikorang | 42.207 31.461 53.341

Olowookere | 25.115 19.582 31.088

Suleiman | 4.963 3.081 7.264

Talam | 6.619 3.716 10.282

Tsega | 13.962 6.815 23.136

Unge | 51.527 42.988 60.021

Sub-total |

D+L pooled % | 19.559 14.067 25.051

---------------------+-----------------------------------------------

AMRO N

Amico | 22.599 13.807 32.827

Cook | 11.238 7.398 15.759

DeMayo | 19.230 13.660 25.498

Gifford | 19.848 11.305 30.086

Golin | 20.134 11.747 30.108

Kleeberger | 30.164 22.344 38.613

Mohammed | 12.790 8.671 17.573

Saberi | 7.438 5.552 9.576

Simoni | 34.310 22.031 47.764

Sub-total |

D+L pooled % | 18.562 13.162 23.962

---------------------+-----------------------------------------------

EURO

Barfod | 16.014 10.201 22.844

Harris | 21.524 12.871 31.689

Moatti | 35.342 23.658 47.992

Morrison | 15.210 7.779 24.569

Riera | 9.150 3.233 17.668

Walsh | 1.567 0.226 4.083

Sub-total |

D+L pooled % | 15.653 6.259 25.046

---------------------+-----------------------------------------------

AMRO S

Harvey | 31.196 23.155 39.853

Monreal | 42.021 32.273 52.094

Remien | 21.641 16.237 27.587

Sub-total |

D+L pooled % | 31.064 19.462 42.665

---------------------+-----------------------------------------------

WPRO

Do | 9.010 6.879 11.396

Gare | 17.696 8.327 29.633

Hansana | 5.509 2.319 9.952

Tran | 1.733 0.395 3.991

Wang | 16.146 8.285 25.999

Weaver | 43.965 31.530 56.797

Sub-total |

D+L pooled % | 12.933 6.734 19.132

----

Overall |

D+L pooled % | 18.586 15.706 21.465

---------------------+-----------------------------------------------

**Problem at time**

Study | % [95% Conf. Interval]

---------------------+-----------------------------------------------

SEARO

Wasti | 11.204 3.998 21.432

Sub-total |

D+L pooled % | 11.204 2.488 19.921

---------------------+-----------------------------------------------

AFRO

Markos | 4.583 1.064 10.388

Oku | 12.950 8.290 18.466

Oku | 1.777 0.405 4.092

Suleiman | 2.056 0.913 3.646

Unge | 3.425 1.007 7.205

Sub-total |

D+L pooled % | 4.091 1.551 6.630

---------------------+-----------------------------------------------

AMRO N

Chesney | 39.421 26.690 52.929

Cook | 11.696 7.780 16.287

Genberg | 23.115 19.542 26.893

Heckman | 15.232 9.559 21.947

Kalichman | 33.097 22.705 44.399

Kerr | 16.970 10.545 24.556

Kleeberger | 16.940 10.815 24.112

Mohammed | 13.720 9.458 18.629

Okonsky | 59.240 49.076 69.019

Saberi | 27.837 24.466 31.340

Sub-total |

D+L pooled % | 24.700 18.209 31.191

---------------------+-----------------------------------------------

EURO

Barfod | 49.609 40.997 58.234

Morrison | 3.588 0.526 9.222

Nieuwkerk | 15.563 9.326 23.045

Sub-total |

D+L pooled % | 22.687 0.000 47.292

---------------------+-----------------------------------------------

WPRO

Do | 33.036 29.378 36.799

Gare | 17.696 8.327 29.633

Tran | 14.676 10.136 19.889

Wang | 9.987 3.946 18.381

Wang | 16.664 10.015 24.602

Sub-total |

D+L pooled % | 18.621 8.561 28.680

---------------------+-----------------------------------------------

Overall |

D+L pooled % | 18.349 13.401 23.297

---------------------+-----------------------------------------------

**Distance to clinic**

Study | % [95% Conf. Interval]

---------------------+-----------------------------------------------

SEARO

Achappa | 5.268 0.784 13.384

Joshi | 20.742 13.197 29.479

Wasti | 66.330 52.635 78.754

Sub-total |

D+L pooled % | 30.223 0.845 59.600

---------------------+-----------------------------------------------

AFRO

Babatunde | 50.000 34.933 65.067

Hardon | 5.195 2.570 8.671

Igwebe | 15.427 8.440 24.048

Koole | 11.298 9.622 13.090

Olowookere | 32.488 26.430 38.856

Pennap | 8.167 4.799 12.328

Salami | 28.664 19.062 39.355

Suleiman | 3.752 2.137 5.797

Tabatabai | 19.256 13.329 25.979

Sub-total |

D+L pooled % | 17.017 11.530 22.504

---------------------+-----------------------------------------------

WPRO

Do | 8.685 6.592 11.035

Hansana | 7.717 3.849 12.776

Sub-total |

D+L pooled % | 8.493 6.504 10.482

---------------------+-----------------------------------------------

Overall |

D+L pooled % | 17.431 12.971 21.891

---------------------+-----------------------------------------------

**Stock outs**

Study | % [95% Conf. Interval]

---------------------+-----------------------------------

SEARO

Vallabhaneni | 5.848 2.270 10.950

Sub-total |

D+L pooled % | 5.848 1.508 10.188

---------------------+-----------------------------------

AFRO

Baltazary | 10.352 6.507 14.966

Byakika-Tusiime | 18.887 12.758 25.900

Eholie | 28.178 22.631 34.079

Essomba | 14.147 10.171 18.655

Ilyaso | 40.640 33.993 47.464

Jean-Baptiste | 0.153 0.140 1.331

Koole | 2.541 1.750 3.474

Nwauche | 19.268 12.044 27.718

Odili | 5.813 3.457 8.732

Okoronkwo | 7.141 3.917 11.234

Tessema | 5.099 1.513 10.638

Uzochukwu | 65.531 57.237 73.374

Sub-total |

D+L pooled % | 16.952 12.359 21.546

---------------------+-----------------------------------

Overall |

D+L pooled % | 16.051 11.696 20.407

---------------------+-----------------------------------

**Sick**

Study | % [95% Conf. Interval]

---------------------+-----------------------------------------------

SEARO

Lal | 22.126 12.691 33.306

Saha | 5.195 2.570 8.671

Vallabhaneni | 5.848 2.270 10.950

Wasti | 37.752 24.833 51.626

Sub-total |

D+L pooled % | 15.366 5.919 24.814

---------------------+-----------------------------------------------

AFRO

Amberbir | 19.883 12.260 28.817

Babatunde | 25.600 13.583 39.885

Byakika-Tusiime | 2.580 0.591 5.914

Dyrehave | 4.343 2.727 6.313

Haberer | 3.809 0.560 9.775

Hardon | 4.700 2.221 8.039

Jean-Baptiste | 7.009 3.620 11.404

Letta | 9.036 4.114 15.626

Markos | 5.904 1.759 12.268

Obirikorang | 3.215 0.470 8.285

Odili | 5.148 2.943 7.925

Oku | 7.396 4.161 11.465

Olowookere | 12.211 8.202 16.888

Shumba | 4.059 0.598 10.398

Suleiman | 2.783 1.419 4.587

Tabatabai | 13.850 8.777 19.859

Tessema | 22.156 14.148 31.376

Uhagaze | 33.716 20.518 48.358

Unge | 7.247 3.454 12.291

Sub-total |

D+L pooled % | 7.721 5.736 9.705

---------------------+-----------------------------------------------

AMRO N

Amico | 18.488 10.486 28.141

Chesney | 27.880 16.653 40.730

Genberg | 18.155 14.915 21.637

Kerr | 16.970 10.545 24.556

Kleeberger | 28.511 20.850 36.853

Mohammed | 15.581 11.054 20.721

Okonsky | 55.979 45.781 65.928

Saberi | 28.604 25.202 32.133

Simoni | 46.078 32.715 59.734

Sub-total |

D+L pooled % | 27.445 20.668 34.222

---------------------+-----------------------------------------------

EURO

Barfod | 42.578 34.166 51.212

Harris | 15.966 8.484 25.256

Morrison | 6.503 1.944 13.472

Nieuwkerk | 22.168 14.811 30.531

Walsh | 15.505 10.303 21.544

Sub-total |

D+L pooled % | 20.295 9.282 31.308

---------------------+-----------------------------------------------

AMRO S

Harvey | 11.535 6.408 17.921

Remien | 37.065 30.532 43.844

Sub-total |

D+L pooled % | 24.243 0.000 49.261

---------------------+-----------------------------------------------

WPRO

Do | 24.432 21.122 27.901

Gare | 7.257 1.714 16.198

Tran | 0.725 0.026 2.364

Weaver | 31.893 20.627 44.352

Sub-total |

D+L pooled % | 15.634 0.000 31.294

---------------------+-----------------------------------------------

Overall |

D+L pooled % | 15.894 13.007 18.780

---------------------+-----------------------------------------------

**Depressed/overwhelmed**

Study | % [95% Conf. Interval]

---------------------+----------------------------------------

SEARO

Achappa | 18.073 8.517 30.220

Bant | 22.525 14.580 31.626

Lal | 7.356 2.209 15.174

Safren | 1.918 0.070 6.186

Saha | 15.593 10.933 20.907

Vallabhaneni | 8.553 4.095 14.441

Sub-total |

D+L pooled % | 11.652 5.214 18.090

---------------------+----------------------------------------

AFRO

Ehlers | 18.095 9.347 28.945

Essomba | 1.739 0.507 3.689

Hardon | 3.213 1.233 6.082

Oku | 20.180 14.442 26.606

Oku | 12.499 8.251 17.480

Olowookere | 20.046 14.999 25.621

Suleiman | 2.299 1.077 3.963

Tsega | 6.599 1.974 13.664

Uhagaze | 17.427 7.701 30.041

Unge | 4.955 1.916 9.312

Sub-total |

D+L pooled % | 9.461 5.881 13.040

---------------------+----------------------------------------

AMRO N

Amico | 60.960 49.580 71.766

Chesney | 18.259 9.053 29.796

DeMayo | 18.046 12.633 24.185

Genberg | 18.155 14.915 21.637

Gifford | 27.203 17.366 38.321

Golin | 17.356 9.552 26.893

Heckman | 12.107 7.051 18.297

Kalichman | 27.462 17.781 38.357

Kerr | 12.382 6.896 19.190

Kleeberger | 29.338 21.595 37.735

Mohammed | 10.464 6.737 14.896

Okonsky | 59.240 49.076 69.019

Saberi | 19.555 16.603 22.686

Sullivan | 3.467 2.326 4.825

Sub-total |

D+L pooled % | 23.130 16.319 29.940

---------------------+----------------------------------------

EURO

Barfod | 35.546 27.512 44.013

Harris | 9.016 3.547 16.661

Morrison | 2.113 0.078 6.802

Sub-total |

D+L pooled % | 15.229 0.000 32.894

---------------------+----------------------------------------

AMRO S

Guimarães | 12.018 5.825 20.050

Harvey | 8.115 3.880 13.721

Remien | 13.183 8.872 18.196

Sub-total |

D+L pooled % | 11.013 7.751 14.275

---------------------+----------------------------------------

WPRO

Do | 9.010 6.879 11.396

Weaver | 31.893 20.627 44.352

Sub-total |

D+L pooled % | 19.680 0.000 42.055

---------------------+----------------------------------------

Overall |

D+L pooled % | 15.533 12.777 18.289

---------------------+----------------------------------------

**Pill burden**

Study | % [95% Conf. Interval]

---------------------+-----------------------------------------------

SEARO

Achappa | 3.103 0.116 9.897

Gokarn | 1.676 0.061 5.416

Lal | 2.390 0.089 7.675

Saha | 5.195 2.570 8.671

Wanchu | 10.166 3.610 19.540

Sub-total |

D+L pooled % | 3.528 1.407 5.649

---------------------+-----------------------------------------------

AFRO

Bello | 13.482 6.242 22.938

Bhat | 24.214 14.594 35.375

Byakika-Tusiime | 5.550 2.337 10.024

Ehlers | 0.433 0.394 3.736

Eholie | 18.008 13.379 23.157

Essomba | 2.904 1.210 5.298

Hardon | 1.724 0.393 3.972

Markos | 1.918 0.070 6.186

Odili | 3.486 1.715 5.851

Okoronkwo | 8.728 5.137 13.157

Oku | 2.098 0.479 4.823

Olowookere | 11.750 7.816 16.360

Suleiman | 5.205 3.274 7.552

Unge | 1.113 0.040 3.615

Uzochukwu | 21.590 15.017 28.987

Sub-total |

D+L pooled % | 6.489 4.276 8.703

---------------------+-----------------------------------------------

AMRO N

Chesney | 14.410 6.288 25.144

Genberg | 8.035 5.825 10.566

Gifford | 19.848 11.305 30.086

Golin | 15.966 8.484 25.256

Kalichman | 19.009 10.799 28.888

Kerr | 8.710 4.173 14.698

Kleeberger | 26.032 18.634 34.189

Mohammed | 10.464 6.737 14.896

Okonsky | 48.369 38.263 58.544

Saberi | 12.193 9.795 14.814

Simoni | 32.349 20.331 45.688

Sub-total |

D+L pooled % | 18.233 13.346 23.119

---------------------+-----------------------------------------------

EURO

Ammassari | 51.875 40.969 62.692

Arrondo-Velasco | 28.999 22.037 36.498

Barfod | 19.920 13.486 27.255

Harris | 2.025 0.074 6.524

Moralejo | 79.239 71.486 86.043

Morrison | 2.113 0.078 6.802

Sodergard | 12.291 8.449 16.735

Sub-total |

D+L pooled % | 27.757 10.883 44.630

---------------------+-----------------------------------------------

AMRO S

Guimarães | 10.751 4.926 18.477

Monreal | 13.293 7.224 20.857

Remien | 12.188 8.040 17.057

Sub-total |

D+L pooled % | 12.107 8.819 15.395

---------------------+-----------------------------------------------

WPRO

Do | 26.217 22.822 29.761

Hansana | 4.036 1.392 7.976

Wang | 6.903 2.068 14.273

Weaver | 33.618 22.134 46.181

Sub-total |

D+L pooled % | 17.144 3.266 31.022

---------------------+-----------------------------------------------

Overall |

D+L pooled % | 13.649 10.971 16.327

---------------------+-----------------------------------------------

**Secrecy/stigma**

Study | % [95% Conf. Interval]

---------------------+----------------------------------------

SEARO

Achappa | 11.681 4.178 22.295

Bant | 2.720 0.396 7.034

Saha | 16.583 11.788 22.013

Vallabhaneni | 38.288 29.499 47.481

Sub-total |

D+L pooled % | 16.953 3.474 30.431

---------------------+----------------------------------------

AFRO

Babatunde | 37.801 23.756 52.980

Baltazary | 1.759 0.401 4.051

Belayneh | 2.639 0.773 5.574

Bello | 11.893 5.137 20.962

Bhat | 36.717 25.422 48.805

Byakika-Tusiime | 2.580 0.591 5.914

Dyrehave | 4.141 2.566 6.072

Haberer | 73.080 61.725 83.078

Hardon | 0.721 0.026 2.353

Igwebe | 56.173 45.307 66.748

Jean-Baptiste | 0.889 0.032 2.894

Koole | 0.977 0.512 1.589

Nwauche | 7.805 3.316 13.976

Oku | 2.703 0.792 5.706

Oku | 6.375 3.393 10.211

Pennap | 9.652 5.976 14.095

Shumba | 9.000 3.177 17.390

Suleiman | 2.541 1.246 4.277

Tadios | 11.578 5.603 19.346

Talam | 5.706 3.031 9.157

Tsega | 53.677 41.833 65.314

Unge | 25.572 18.495 33.363

Uzochukwu | 7.950 3.968 13.153

Sub-total |

D+L pooled % | 11.371 8.932 13.809

---------------------+----------------------------------------

AMRO N

Cook | 0.669 0.024 2.181

Genberg | 4.067 2.520 5.964

Gifford | 71.326 60.079 81.375

Golin | 3.438 0.503 8.847

Kalichman | 9.143 3.599 16.887

Kleeberger | 29.338 21.595 37.735

Mohammed | 24.418 18.921 30.372

Nyaku | 8.293 5.839 11.131

Simoni | 22.541 12.245 34.894

Sullivan | 0.424 0.096 0.983

Sub-total |

D+L pooled % | 13.929 9.837 18.022

---------------------+----------------------------------------

EURO

Ammassari | 9.366 4.004 16.671

Barfod | 17.576 11.501 24.623

Blanco | 2.247 0.514 5.161

Harris | 40.971 29.953 52.470

Moatti | 21.545 12.018 32.947

Nieuwkerk | 19.337 12.418 27.365

Riera | 30.830 19.880 43.001

Spire | 7.078 3.199 12.327

Sub-total |

D+L pooled % | 17.609 9.769 25.448

---------------------+----------------------------------------

AMRO S

Gir | 20.063 14.190 26.667

Guimarães | 10.751 4.926 18.477

Monreal | 40.957 31.268 51.015

Sub-total |

D+L pooled % | 23.493 8.449 38.536

---------------------+----------------------------------------

WPRO

Cummings | 42.981 30.509 55.924

Do | 9.821 7.599 12.294

Gare | 30.203 18.174 43.806

Wang | 5.545 1.926 10.883

Weaver | 6.006 1.407 13.502

Sub-total |

D+L pooled % | 16.271 8.108 24.435

---------------------+----------------------------------------

Overall |

D+L pooled % | 13.610 11.915 15.304

---------------------+----------------------------------------

**Alcohol/substance misuse**

Study | % [95% Conf. Interval]

---------------------+-----------------------------------------------

SEARO

Achappa | 18.073 8.517 30.220

Naik | 38.635 27.331 50.598

Safren | 7.223 2.529 14.071

Vallabhaneni | 2.230 0.323 5.785

Wasti | 11.204 3.998 21.432

Sub-total |

D+L pooled % | 14.382 4.451 24.312

---------------------+-----------------------------------------------

AFRO

Hardon | 4.700 2.221 8.039

Suleiman | 2.299 1.077 3.963

Sub-total |

D+L pooled % | 3.153 0.900 5.407

---------------------+-----------------------------------------------

AMRO N

Amico | 14.377 7.332 23.288

Cook | 28.669 22.872 34.842

Fredericksen | 15.098 6.103 27.188

Genberg | 2.876 1.598 4.513

Golin | 13.187 6.418 21.910

Kalichman | 10.553 4.533 18.698

Kerr | 13.299 7.607 20.282

Mohammed | 9.068 5.607 13.261

Simoni | 28.426 17.009 41.458

Sub-total |

D+L pooled % | 14.519 8.160 20.878

---------------------+-----------------------------------------------

EURO

Barfod | 42.578 34.166 51.212

Walsh | 13.606 8.723 19.372

Sub-total |

D+L pooled % | 27.893 0.000 56.282

---------------------+-----------------------------------------------

WPRO

Do | 12.906 10.377 15.666

Gare | 9.349 2.839 19.091

Tran | 2.232 0.652 4.723

Sub-total |

D+L pooled % | 8.031 0.000 16.392

---------------------+-----------------------------------------------

Overall |

D+L pooled % | 12.923 9.733 16.114

---------------------+-----------------------------------------------

**Lack of food**

Study | % [95% Conf. Interval]

---------------------+-----------------------------------------------

AFRO

Dyrehave | 7.575 5.412 10.068

Hardon | 5.691 2.927 9.296

Koole | 29.750 27.276 32.284

Mbopi-Kéou | 7.756 4.262 12.181

Obirikorang | 9.731 4.166 17.296

Oku | 5.719 2.712 9.749

Oku | 11.478 7.407 16.303

Sub-total |

D+L pooled % | 11.141 3.140 19.141

---------------------+-----------------------------------------------

EURO

Arrondo-Velasco | 8.330 4.459 13.266

Sub-total |

D+L pooled % | 8.330 3.927 12.734

---------------------+-----------------------------------------------

AMRO S

Harvey | 26.922 19.307 35.293

Sub-total |

D+L pooled % | 26.922 18.929 34.915

---------------------+-----------------------------------------------

WPRO

Gare | 15.611 6.846 27.109

Wang | 11.611 6.084 18.624

Sub-total |

D+L pooled % | 12.719 7.387 18.050

---------------------+-----------------------------------------------

Overall |

D+L pooled % | 12.589 6.528 18.650

---------------------+-----------------------------------------------

**Palatability**

Study | % [95% Conf. Interval]

---------------------+-----------------------------------------------

AFRO

Talam | 7.076 4.066 10.838

Sub-total |

D+L pooled % | 7.076 3.690 10.462

---------------------+-----------------------------------------------

EURO

Arrondo-Velasco | 8.997 4.962 14.081

Barfod | 23.827 16.875 31.562

Moralejo | 2.951 0.678 6.752

Walsh | 16.771 11.372 22.976

Sub-total |

D+L pooled % | 12.700 4.146 21.254

---------------------+-----------------------------------------------

WPRO

Wang | 14.643 8.407 22.246

Sub-total |

D+L pooled % | 14.643 7.724 21.562

---------------------+-----------------------------------------------

Overall |

D+L pooled % | 11.780 6.313 17.248

---------------------+-----------------------------------------------

**Felt good**

Study | % [95% Conf. Interval]

---------------------+-----------------------------------------------

SEARO

Achappa | 7.412 1.753 16.528

Gokarn | 9.763 4.457 16.839

Joshi | 2.633 0.383 6.813

Lal | 2.390 0.089 7.675

Safren | 5.904 1.759 12.268

Saha | 3.213 1.233 6.082

Vallabhaneni | 1.313 0.048 4.259

Sub-total |

D+L pooled % | 3.395 1.640 5.150

---------------------+-----------------------------------------------

AFRO

Babatunde | 20.718 9.867 34.292

Bhat | 10.143 4.011 18.656

Hardon | 1.724 0.393 3.972

Igwebe | 16.662 9.398 25.501

Koole | 3.714 2.747 4.819

Markos | 3.257 0.476 8.391

Okoronkwo | 16.137 11.253 21.707

Olowookere | 26.497 20.851 32.560

Suleiman | 6.900 4.660 9.541

Unge | 1.889 0.273 4.913

Sub-total |

D+L pooled % | 9.206 5.855 12.556

---------------------+-----------------------------------------------

AMRO N

Amico | 1.997 0.073 6.436

Genberg | 12.004 9.315 14.982

Saberi | 17.101 14.311 20.085

Simoni | 28.426 17.009 41.458

Sub-total |

D+L pooled % | 13.495 5.470 21.521

---------------------+-----------------------------------------------

EURO

Barfod | 28.515 21.054 36.619

Harris | 9.016 3.547 16.661

Morrison | 6.503 1.944 13.472

Sub-total |

D+L pooled % | 14.455 1.853 27.056

---------------------+-----------------------------------------------

AMRO S

Harvey | 8.970 4.492 14.791

Remien | 18.159 13.150 23.775

Sub-total |

D+L pooled % | 13.540 4.535 22.545

---------------------+-----------------------------------------------

WPRO

Do | 4.951 3.380 6.803

Weaver | 21.545 12.018 32.947

Sub-total |

D+L pooled % | 12.412 0.000 28.592

---------------------+-----------------------------------------------

Overall |

D+L pooled % | 9.307 7.169 11.445

---------------------+-----------------------------------------------

**Patient report barriers to adherence: Children**

**Forgot**

Study | % [95% Conf. Interval]

---------------------+-----------------------------------------------

SEARO

Mehta | 16.031 5.054 31.616

Seth | 10.946 3.353 22.169

Sub-total |

D+L pooled % | 12.645 4.968 20.322

---------------------+-----------------------------------------------

AFRO

Aderemi-Williams | 45.120 30.352 60.342

Arage | 28.946 20.312 38.428

Biadgilign | 24.032 13.514 36.456

Biressaw | 35.914 25.234 47.352

Dachew | 52.353 41.762 62.838

Elise | 40.786 25.885 56.611

Iroha | 8.257 1.261 20.541

Ugwu | 55.114 44.686 65.319

Sub-total |

D+L pooled % | 36.102 24.340 47.864

---------------------+-----------------------------------------------

AMRO N

Marhefka | 8.630 2.610 17.687

Marhefka | 23.116 15.160 32.179

Sub-total |

D+L pooled % | 15.733 1.539 29.927

---------------------+-----------------------------------------------

AMRO S

White | 35.521 21.285 51.208

Sub-total |

D+L pooled % | 35.521 20.560 50.482

---------------------+-----------------------------------------------

Overall |

D+L pooled % | 29.249 20.102 38.396

---------------------+-----------------------------------------------

**Change to routine**

Study | % [95% Conf. Interval]

---------------------+-----------------------------------------------

AFRO

Aderemi-Williams | 15.835 6.422 28.423

Elise | 11.810 3.635 23.814

Sub-total |

D+L pooled % | 13.649 6.213 21.084

---------------------+-----------------------------------------------

AMRO N

Buchanan | 30.403 20.520 41.300

Marhefka | 39.247 29.612 49.324

Sub-total |

D+L pooled % | 34.984 26.323 43.645

---------------------+-----------------------------------------------

AMRO S

White | 35.521 21.285 51.208

Sub-total |

D+L pooled % | 35.521 20.560 50.482

---------------------+-----------------------------------------------

Overall |

D+L pooled % | 26.325 15.268 37.382

---------------------+-----------------------------------------------

**Secrecy/stigma**

Study | % [95% Conf. Interval]

---------------------+-----------------------------------------------

SEARO

Seth | 18.277 8.105 31.397

Sub-total |

D+L pooled % | 18.277 6.631 29.923

---------------------+-----------------------------------------------

AFRO

Aderemi-Williams | 64.639 49.552 78.365

Dachew | 5.279 1.568 11.003

Eticha | 31.809 17.273 48.441

Iroha | 11.615 2.824 25.268

Ugwu | 0.285 0.261 2.472

Sub-total |

D+L pooled % | 20.751 7.221 34.280

---------------------+-----------------------------------------------

AMRO N

Buchanan | 33.106 22.913 44.173

Sub-total |

D+L pooled % | 33.106 22.477 43.736

---------------------+-----------------------------------------------

Overall |

D+L pooled % | 22.325 10.189 34.460

---------------------+-----------------------------------------------

**Travel**

Study | % [95% Conf. Interval]

---------------------+-----------------------------------------------

SEARO

Mehta | 26.767 12.316 44.369

Sub-total |

D+L pooled % | 26.767 10.740 42.794

---------------------+-----------------------------------------------

AFRO

Eticha | 7.505 1.139 18.771

Iroha | 24.981 11.404 41.729

Ugwu | 25.566 17.049 35.146

Sub-total |

D+L pooled % | 18.821 5.864 31.777

---------------------+-----------------------------------------------

AMRO N

Buchanan | 27.700 18.168 38.385

Marhefka | 2.805 0.105 8.969

Marhefka | 13.436 7.305 21.073

Sub-total |

D+L pooled % | 13.991 0.850 27.132

---------------------+-----------------------------------------------

AMRO S

White | 27.622 14.766 42.721

Sub-total |

D+L pooled % | 27.622 13.644 41.600

---------------------+-----------------------------------------------

Overall |

D+L pooled % | 18.531 10.255 26.808

---------------------+-----------------------------------------------

**Palatability**

Study | % [95% Conf. Interval]

---------------------+-----------------------------------------------

SEARO

Seth | 6.040 0.905 15.262

Sub-total |

D+L pooled % | 6.040 0.000 13.219

---------------------+-----------------------------------------------

AFRO

Arage | 2.606 0.379 6.743

Davies | 22.041 9.933 37.278

Ugwu | 3.958 0.915 9.003

Sub-total |

D+L pooled % | 5.766 0.000 11.564

---------------------+-----------------------------------------------

AMRO N

Marhefka | 10.558 3.756 20.255

van Dyke | 18.073 8.517 30.220

Sub-total |

D+L pooled % | 13.455 6.285 20.624

---------------------+-----------------------------------------------

EURO

Gibb | 73.488 62.262 83.354

Sub-total |

D+L pooled % | 73.488 62.942 84.034

---------------------+-----------------------------------------------

AMRO S

White | 14.449 5.240 27.222

Sub-total |

D+L pooled % | 14.449 3.458 25.440

---------------------+-----------------------------------------------

Overall |

D+L pooled % | 18.396 6.634 30.157

---------------------+-----------------------------------------------

**Distance to clinic**

Study | % [95% Conf. Interval]

---------------------+-----------------------------------------------

SEARO

Mehta | 51.787 33.578 69.753

Sub-total |

D+L pooled % | 51.787 33.699 69.875

---------------------+-----------------------------------------------

AFRO

Arage | 19.471 12.177 27.995

Biadgilign | 6.699 1.577 15.000

Biressaw | 3.487 0.511 8.968

Sub-total |

D+L pooled % | 9.401 0.584 18.219

---------------------+-----------------------------------------------

Overall |

D+L pooled % | 17.456 4.662 30.249

---------------------+-----------------------------------------------

**Busy**

Study | % [95% Conf. Interval]

---------------------+-----------------------------------------------

SEARO

Seth | 32.921 19.554 47.876

Sub-total |

D+L pooled % | 32.921 18.760 47.082

---------------------+-----------------------------------------------

AFRO

Aderemi-Williams | 30.481 17.517 45.260

Eticha | 4.421 0.170 13.927

Ugwu | 8.514 3.628 15.205

Vreeman | 12.943 7.396 19.758

Sub-total |

D+L pooled % | 12.127 4.705 19.549

---------------------+-----------------------------------------------

AMRO N

Buchanan | 18.238 10.337 27.782

Marhefka | 4.762 0.705 12.139

Marhefka | 18.814 11.568 27.345

Sub-total |

D+L pooled % | 13.554 3.595 23.514

---------------------+-----------------------------------------------

AMRO S

White | 27.622 14.766 42.721

Sub-total |

D+L pooled % | 27.622 13.644 41.600

---------------------+-----------------------------------------------

Overall |

D+L pooled % | 15.747 9.890 21.604

---------------------+-----------------------------------------------

**Stock outs**

Study | % [95% Conf. Interval]

---------------------+-----------------------------------------------

AFRO

Elise | 48.684 33.124 64.375

Sub-total |

D+L pooled % | 48.684 33.058 64.309

---------------------+-----------------------------------------------

AMRO N

Marhefka | 2.805 0.105 8.969

Marhefka | 2.662 0.387 6.885

Sub-total |

D+L pooled % | 2.712 0.091 5.332

---------------------+-----------------------------------------------

AMRO S

White | 19.720 8.799 33.677

Sub-total |

D+L pooled % | 19.720 7.282 32.159

---------------------+-----------------------------------------------

Overall |

D+L pooled % | 15.384 3.967 26.802

---------------------+-----------------------------------------------

**Ran out of pills**

Study | % [95% Conf. Interval]

---------------------+-----------------------------------------------

SEARO

Mehta | 33.917 17.870 52.145

Sub-total |

D+L pooled % | 33.917 16.780 51.055

---------------------+-----------------------------------------------

AFRO

Aderemi-Williams | 6.040 0.905 15.262

Arage | 13.153 7.145 20.647

Biadgilign | 27.880 16.653 40.730

Biressaw | 2.054 0.076 6.614

Elise | 3.839 0.146 12.159

Eticha | 4.421 0.170 13.927

Iroha | 55.003 37.261 72.111

Ugwu | 18.747 11.331 27.522

Sub-total |

D+L pooled % | 13.849 6.519 21.178

---------------------+-----------------------------------------------

AMRO N

Marhefka | 4.762 0.705 12.139

Marhefka | 15.588 8.974 23.617

Sub-total |

D+L pooled % | 9.923 0.000 20.521

---------------------+-----------------------------------------------

AMRO S

White | 30.255 16.884 45.605

Sub-total |

D+L pooled % | 30.255 15.895 44.616

---------------------+-----------------------------------------------

Overall |

D+L pooled % | 15.312 9.303 21.321

---------------------+-----------------------------------------------

**Depressed/overwhelmed**

Study | % [95% Conf. Interval]

---------------------+-----------------------------------------------

AFRO

Biadgilign | 6.699 1.577 15.000

Eticha | 25.743 12.512 41.771

Sub-total |

D+L pooled % | 15.066 0.000 33.591

---------------------+-----------------------------------------------

AMRO S

White | 17.085 6.969 30.501

Sub-total |

D+L pooled % | 17.085 5.319 28.851

---------------------+-----------------------------------------------

Overall |

D+L pooled % | 15.071 3.880 26.262

---------------------+-----------------------------------------------

**Asleep**

Study | % [95% Conf. Interval]

---------------------+-----------------------------------------------

SEARO

Seth | 3.558 0.135 11.298

Sub-total |

D+L pooled % | 3.558 0.000 9.140

---------------------+-----------------------------------------------

AFRO

Aderemi-Williams | 25.600 13.583 39.885

Biadgilign | 25.956 15.069 38.608

Biressaw | 7.732 2.713 15.027

Iroha | 24.981 11.404 41.729

Ugwu | 9.652 4.404 16.654

Sub-total |

D+L pooled % | 16.982 8.963 25.002

---------------------+-----------------------------------------------

AMRO S

White | 14.449 5.240 27.222

Sub-total |

D+L pooled % | 14.449 3.458 25.440

---------------------+-----------------------------------------------

Overall |

D+L pooled % | 14.078 7.730 20.426

---------------------+-----------------------------------------------

**Sick**

Study | % [95% Conf. Interval]

---------------------+-----------------------------------------------

AFRO

Aderemi-Williams | 15.835 6.422 28.423

Arage | 3.666 0.846 8.353

Biadgilign | 2.805 0.105 8.969

Biressaw | 11.963 5.506 20.468

Eticha | 10.558 2.549 23.114

Iroha | 4.864 0.189 15.258

Ugwu | 9.652 4.404 16.654

Sub-total |

D+L pooled % | 7.027 3.698 10.355

---------------------+-----------------------------------------------

AMRO N

Marhefka | 4.762 0.705 12.139

Sub-total |

D+L pooled % | 4.762 0.000 10.479

---------------------+-----------------------------------------------

AMRO S

White | 14.449 5.240 27.222

Sub-total |

D+L pooled % | 14.449 3.458 25.440

---------------------+-----------------------------------------------

Overall |

D+L pooled % | 7.060 4.176 9.944

---------------------+-----------------------------------------------

**Toxicity**

Study | % [95% Conf. Interval]

---------------------+-----------------------------------------------

SEARO

Seth | 0.614 0.556 5.262

Sub-total |

D+L pooled % | 0.614 0.000 2.967

---------------------+-----------------------------------------------

AFRO

Arage | 4.723 1.398 9.872

Eticha | 16.639 6.100 31.024

Sub-total |

D+L pooled % | 9.181 0.000 20.482

---------------------+-----------------------------------------------

AMRO N

Marhefka | 4.762 0.705 12.139

Sub-total |

D+L pooled % | 4.762 0.000 10.479

---------------------+-----------------------------------------------

AMRO S

White | 17.085 6.969 30.501

Sub-total |

D+L pooled % | 17.085 5.319 28.851

---------------------+-----------------------------------------------

Overall |

D+L pooled % | 6.113 1.247 10.979

---------------------+-----------------------------------------------

**Pill burden**

Study | % [95% Conf. Interval]

---------------------+-----------------------------------------------

SEARO

Seth | 0.614 0.556 5.262

Sub-total |

D+L pooled % | 0.614 0.000 2.967

---------------------+-----------------------------------------------

AFRO

Arage | 4.723 1.398 9.872

Eticha | 16.639 6.100 31.024

Ugwu | 7.376 2.882 13.725

Sub-total |

D+L pooled % | 7.169 2.538 11.799

---------------------+-----------------------------------------------

AMRO N

Marhefka | 6.699 1.577 15.000

Sub-total |

D+L pooled % | 6.699 0.000 13.410

---------------------+-----------------------------------------------

AMRO S

White | 9.168 2.193 20.236

Sub-total |

D+L pooled % | 9.168 0.147 18.189

---------------------+-----------------------------------------------

Overall |

D+L pooled % | 5.785 1.948 9.621

---------------------+-----------------------------------------------

**Patient report barriers to adherence: Adolescents**

**Forgot**

Study | % [95% Conf. Interval]

---------------------+-----------------------------------------------

AFRO

Angevine | 40.274 25.051 56.526

Mavhu | 92.029 85.736 96.611

Sub-total |

D+L pooled % | 66.700 15.992 117.408

---------------------+-----------------------------------------------

AMRO N

MacDonnell | 73.505 69.490 77.335

Murphy | 65.653 56.761 74.023

Sub-total |

D+L pooled % | 70.559 63.107 78.011

---------------------+-----------------------------------------------

AMRO S

Crozatti | 40.094 30.999 49.546

Sub-total |

D+L pooled % | 40.094 30.820 49.367

---------------------+-----------------------------------------------

Overall |

D+L pooled % | 63.133 46.311 79.955

---------------------+-----------------------------------------------

**Travel**

Study | % [95% Conf. Interval]

---------------------+-----------------------------------------------

AFRO

Angevine | 31.936 17.931 47.852

Mavhu | 47.340 37.370 57.419

Sub-total |

D+L pooled % | 40.680 25.724 55.637

---------------------+-----------------------------------------------

Overall |

D+L pooled % | 40.680 25.724 55.637

---------------------+-----------------------------------------------

**Pill burden**

Study | % [95% Conf. Interval]

---------------------+-----------------------------------------------

AMRO N

Murphy | 36.956 28.400 45.946

Sub-total |

D+L pooled % | 36.956 28.183 45.729

---------------------+-----------------------------------------------

Overall |

D+L pooled % | 36.956 28.183 45.729

---------------------+-----------------------------------------------

**Asleep**

Study | % [95% Conf. Interval]

---------------------+-----------------------------------------------

AFRO

Angevine | 23.597 11.368 38.618

Sub-total |

D+L pooled % | 23.597 9.972 37.222

---------------------+-----------------------------------------------

AMRO N

Murphy | 48.261 39.208 57.372

Sub-total |

D+L pooled % | 48.261 39.179 57.343

---------------------+-----------------------------------------------

Overall |

D+L pooled % | 36.473 12.327 60.620

---------------------+-----------------------------------------------

**Distance to clinic**

Study | % [95% Conf. Interval]

---------------------+-----------------------------------------------

AFRO

Mavhu | 35.637 26.314 45.546

Sub-total |

D+L pooled % | 35.637 26.021 45.253

---------------------+-----------------------------------------------

Overall |

D+L pooled % | 35.637 26.021 45.253

---------------------+-----------------------------------------------

**Palatability**

Study | % [95% Conf. Interval]

---------------------+-----------------------------------------------

AMRO N

MacDonnell | 20.515 17.043 24.221

Murphy | 33.478 25.176 42.330

Sub-total |

D+L pooled % | 26.387 13.741 39.034

---------------------+-----------------------------------------------

AMRO S

Crozatti | 35.377 26.593 44.688

Sub-total |

D+L pooled % | 35.377 26.329 44.424

---------------------+-----------------------------------------------

Overall |

D+L pooled % | 29.176 18.398 39.954

---------------------+-----------------------------------------------

**Alcohol/substance misuse**

Study | % [95% Conf. Interval]

---------------------+-----------------------------------------------

AMRO N

Rao | 28.827 13.387 47.357

Sub-total |

D+L pooled % | 28.827 11.842 45.812

---------------------+---------------------------------------------------

Overall |

D+L pooled % | 28.827 11.842 45.812

---------------------+-----------------------------------------------

**Busy**

Study | % [95% Conf. Interval]

---------------------+-----------------------------------------------

AFRO

Angevine | 18.035 7.388 32.062

Sub-total |

D+L pooled % | 18.035 5.698 30.372

---------------------+-----------------------------------------------

AMRO N

MacDonnell | 15.567 12.481 18.925

Murphy | 47.391 38.359 56.510

Sub-total |

D+L pooled % | 31.185 0.003 62.367

---------------------+-----------------------------------------------

Overall |

D+L pooled % | 26.927 6.104 47.750

---------------------+-----------------------------------------------

**Felt good**

Study | % [95% Conf. Interval]

---------------------+-----------------------------------

AMRO N

Murphy | 26.521 18.888 34.935

Sub-total |

D+L pooled % | 26.521 18.497 34.544

---------------------+-----------------------------------

Overall |

D+L pooled % | 26.521 18.497 34.544

---------------------+-----------------------------------

**Depressed/overwhelmed**

Study | % [95% Conf. Interval]

---------------------+-----------------------------------------------

AMRO N

Murphy | 25.651 18.119 33.994

Sub-total |

D+L pooled % | 25.651 17.714 33.588

---------------------+-----------------------------------------------

Overall |

D+L pooled % | 25.651 17.714 33.588

---------------------+-----------------------------------------------

**Ran out of pills**

Study | % [95% Conf. Interval]

---------------------+-----------------------------------

AMRO N

MacDonnell | 20.515 17.043 24.221

Murphy | 33.478 25.176 42.330

Sub-total |

D+L pooled % | 26.387 13.741 39.034

---------------------+-----------------------------------

AMRO S

Crozatti | 24.055 16.436 32.611

Sub-total |

D+L pooled % | 24.055 15.967 32.143

---------------------+-----------------------------------

Overall |

D+L pooled % | 25.282 17.807 32.756

---------------------+-----------------------------------

**Sick**

Study | % [95% Conf. Interval]

---------------------+-----------------------------------

AMRO N

MacDonnell | 12.474 9.686 15.559

Murphy | 28.260 20.438 36.806

Sub-total |

D+L pooled % | 19.886 4.445 35.327

---------------------+-----------------------------------

Overall |

D+L pooled % | 19.886 4.445 35.327

---------------------+-----------------------------------

**Avoid side effects**

Study | % [95% Conf. Interval]

---------------------+-----------------------------------------------

AMRO N

Murphy | 19.563 12.860 27.280

Sub-total |

D+L pooled % | 19.563 12.354 26.773

---------------------+-----------------------------------------------

Overall |

D+L pooled % | 19.563 12.354 26.773

---------------------+-----------------------------------------------

**Lack of food**

Study | % [95% Conf. Interval]

---------------------+-----------------------------------

AFRO

Angevine | 18.035 7.388 32.062

Sub-total |

D+L pooled % | 18.035 5.698 30.372

---------------------+-----------------------------------

Overall |

D+L pooled % | 18.035 5.698 30.372

---------------------+-----------------------------------

**Toxicity**

Study | % [95% Conf. Interval]

---------------------+-----------------------------------------------

AFRO

Angevine | 12.467 3.852 25.052

Sub-total |

D+L pooled % | 12.467 1.867 23.067

---------------------+-----------------------------------------------

AMRO N

Murphy | 12.605 7.196 19.261

Sub-total |

D+L pooled % | 12.605 6.573 18.638

---------------------+-----------------------------------------------

Overall |

D+L pooled % | 12.572 7.329 17.815

---------------------+-----------------------------------------------

**Stock outs**

Study | % [95% Conf. Interval]

---------------------+----------------------------------------

AMRO N

MacDonnell | 11.237 8.583 14.197

Sub-total |

D+L pooled % | 11.237 8.430 14.044

---------------------+----------------------------------------

Overall |

D+L pooled % | 11.237 8.430 14.044

---------------------+----------------------------------------
