# Supplementary material for: Patient-Reported Barriers to Adherence to Antiretroviral Therapy: A Systematic Review and Meta-Analysis
Source: PLoS Med. 2016 Nov 29;13(11):e1002183. doi: 10.1371/journal.pmed.1002183 (PMC5127502; doi:10.1371/journal.pmed.1002183)
Supplement: S1 Text — (DOCX) [file pmed.1002183.s003.docx]

**Systematic review protocol**

**Self-reported barriers to adherence to antiretroviral therapy among people living with HIV: a global review**

**Introduction**

Adherence to antiretroviral therapy (ART) is a primary determinant of HIV virological suppression and risk of HIV transmission, disease progression, and death.(1-3) Sub-optimal ART adherence is a major challenge in all regions and is associated with a diversity of patient and programme-related challenges. Individual factors may include forgetting doses, being away from home, changes in daily routines, depression or other illness, limited understanding of treatment benefits, a lack of interest or desire to take the medicines and substance or alcohol use. Adherence to ART may also be challenging in the absence of supportive environments for people living with HIV and due to HIV-related stigma and discrimination. Medication-related factors may include adverse drug events, the complexity of dosing regimens, high pill burden and dietary restrictions.(4, 5)

While these and other risk factors have been described by individual studies, there remains a lack of overall understanding of the most important contributors to poor ART adherence. Such an understanding is critical to help direct research and care efforts towards solving the most common problems faced by patients.

This systematic review aims to summarize and rank barriers to adherence to antiretroviral therapy among people living with HIV.

**Methods**

This review will follow the PRISMA standards for conducting systematic reviews and meta-analyses.

**Inclusions and exclusions**

***Inclusion criteria***

- Any study design that provides quantitative data on patient-reported adherence barriers in ≥50 adults or ≥20 children
- No language, geographical or age limits will be applied

***Exclusion criteria***

- Interventional studies of adherence interventions
- Studies providing ART as mono or bi-therapy
- ARV interventions other than combination antiretroviral therapy: PMTCT, PrEP, PEP
- Studies reporting barriers among adherent and non-adherent populations where the cannot be disaggregated

**Search strategy**

Using a highly sensitive search strategy (See below) the following databases will be screened:

**Databases**

- PubMed
- EMBASE
- Web of Science

PsychINFO

**Conference abstracts**

- International AIDS society (from 2012)
- IAPAC (from 2012)
- ESPACOMP (from 2012)
- CROI (from 2014)

**Search strategy for PubMed**

| 1 | Antiretroviral Therapy, Highly Active[MeSH] OR Anti-Retroviral Agents[MeSH] OR Antiviral Agents[MeSH:NoExp] OR ((anti) AND (hiv[tw])) OR antiretroviral*[tw] OR ((anti) AND (retroviral*[tw])) OR HAART[tw] OR ((anti) AND (acquired immunodeficiency[tw])) OR ((anti) AND (acquired immunedeficiency[tw])) OR ((anti) AND (acquired immuno-deficiency[tw])) OR ((anti) AND (acquired immune-deficiency[tw])) OR ((anti) AND (acquired immun*) AND (deficiency[tw])) |
| --- | --- |
| 2 | Compliance[tw] OR adherence[MeSH:NoExp] OR adherence[tw] OR comply[MeSH:NoExp] OR comply[tw] OR adhere[tw] |
| 3 | reason[tw] OR reasons[tw] OR cause[tw] OR causes[tw] OR determinant[tw] OR determinants[tw] OR factor[tw] OR factors[tw] OR barrier [tw] OR barriers[tw] OR (Away from home[tw]) OR forgot[tw] OR (Change in daily routine[tw]) OR (asleep[tw]) OR (asleep[tw]) OR (slept[tw]) OR busy[tw] OR sick[tw] OR (side effect[tw]) OR depressed[tw] OR (felt good[tw]) OR toxic[tw] |

**Data extraction**

The number of individuals reporting experiencing each barrier to adherence will be reported, based on the self-reported Adult AIDS Clinical Trial Groups (AACTG) adherence instrument,(6) with additional categories included to reflect challenges common in resource-limited settings.

Where possible, data will be extracted only on patients who are recorded as non-adherent, but if this is not possible data on barriers to ART adherence for the whole cohort – including those who are adherent despite barriers - will be reported.

The following criteria will be used as indicative of risk of bias:

- Retrospective study design
- Sampling bias

**Data analysis**

The proportion of patients reporting each barrier will be calculated as a proportion of all patients reporting barriers, and data pooled across studies using random-effects meta-analysis. Results will be displayed graphically and hierarchically in forest plots.

Data will be disaggregated by age, and the influence of the following covariates assessed in subgroup analysis/meta-regression:

Date of study:

- toxicity/side effects
- Secrecy/stigma
- Pill burden
- Palatability (children)
- Distance to clinic

All analyses will be conducted in STATA version 13.0.

**References**

1. Bangsberg DR, Perry S, Charlebois ED, Clark RA, Roberston M, Zolopa AR, et al. Non-adherence to highly active antiretroviral therapy predicts progression to AIDS. Aids. 2001;15(9):1181-3.

2. Nachega JB, Hislop M, Dowdy DW, Chaisson RE, Regensberg L, Maartens G. Adherence to nonnucleoside reverse transcriptase inhibitor-based HIV therapy and virologic outcomes. Annals of internal medicine. 2007;146(8):564-73.

3. Wood E, Hogg RS, Yip B, Harrigan PR, O'Shaughnessy MV, Montaner JS. Effect of medication adherence on survival of HIV-infected adults who start highly active antiretroviral therapy when the CD4+ cell count is 0.200 to 0.350 x 10(9) cells/L. Annals of internal medicine. 2003;139(10):810-6.

4. Mills EJ, Nachega JB, Bangsberg DR, Singh S, Rachlis B, Wu P, et al. Adherence to HAART: a systematic review of developed and developing nation patient-reported barriers and facilitators. PLoS medicine. 2006;3(11):e438.

5. Martin S, Elliott-DeSorbo DK, Wolters PL, Toledo-Tamula MA, Roby G, Zeichner S, et al. Patient, caregiver and regimen characteristics associated with adherence to highly active antiretroviral therapy among HIV-infected children and adolescents. The Pediatric infectious disease journal. 2007;26(1):61-7.

6. Chesney MA, Ickovics JR, Chambers DB, Gifford AL, Neidig J, Zwickl B, et al. Self-reported adherence to antiretroviral medications among participants in HIV clinical trials: the AACTG adherence instruments. Patient Care Committee & Adherence Working Group of the Outcomes Committee of the Adult AIDS Clinical Trials Group (AACTG). AIDS Care. 2000;12(3):255-66.
